# Role of an unclassified *Lachnospiraceae* in the pathogenesis of type 2 diabetes: a longitudinal study of the urine microbiome and metabolites

**DOI:** 10.1038/s12276-022-00816-x

**Published:** 2022-08-05

**Authors:** Kangjin Kim, Sanghun Lee, Sang-Chul Park, Nam-Eun Kim, Chol Shin, Seung Ku Lee, Youngae Jung, Dankyu Yoon, Hyeonjeong Kim, Sanghyun Kim, Geum-Sook Hwang, Sungho Won

**Affiliations:** 1 Department of Public Health Science, Graduate School of Public Health, Seoul National University, Seoul, South Korea; 2 Department of Medical Consilience, Graduate School, Dankook University, Seoul, South Korea; 3 Brigham and Women’s Hospital, Harvard Medical School, Boston, MA USA; 4 Division of Pulmonary Sleep and Critical Care Medicine, Department of Internal Medicine, Korea University Ansan Hospital, Ansan, South Korea; 5 Institute of Human Genomic Study, College of Medicine, Korea University Ansan Hospital, Ansan, South Korea; 6 Integrated Metabolomics Research Group, Western Seoul Center, Korea Basic Science Institute, Seoul, South Korea; 7 Division of Allergy and Chronic Respiratory Diseases, Center for Biomedical Sciences, National Institute of Health, Korea Center for Diseases Control and Prevention, Osong, Cheongju, South Korea; 8 Korea Medical Institute, Seoul, South Korea; 9 Interdisciplinary Program for Bioinformatics, College of Natural Science, Seoul National University, Seoul, South Korea; 10 Institute of Health and Environment, Seoul National University, Seoul, South Korea

**Keywords:** Bacterial genetics, Calcium and phosphate metabolic disorders

## Abstract

Recent investigations have revealed that the human microbiome plays an essential role in the occurrence of type 2 diabetes (T2D). However, despite the importance of understanding the involvement of the microbiota throughout the body in T2D, most studies have focused specifically on the intestinal microbiota. Extracellular vesicles (EVs) have been recently found to provide important evidence regarding the mechanisms of T2D pathogenesis, as they act as key messengers between intestinal microorganisms and the host. Herein, we explored microorganisms potentially associated with T2D by tracking changes in microbiota-derived EVs from patient urine samples collected three times over four years. Mendelian randomization analysis was conducted to evaluate the causal relationships among microbial organisms, metabolites, and clinical measurements to provide a comprehensive view of how microbiota can influence T2D. We also analyzed EV-derived metagenomic (*N* = 393), clinical (*N* = 5032), genomic (*N* = 8842), and metabolite (*N* = 574) data from a prospective longitudinal Korean community-based cohort. Our data revealed that *GU174097_g*, an unclassified *Lachnospiraceae*, was associated with T2D (*β* = −189.13; *p* = 0.00006), and it was associated with the ketone bodies acetoacetate and 3-hydroxybutyrate (*r* = −0.0938 and −0.0829, respectively; *p* = 0.0022 and 0.0069, respectively). Furthermore, a causal relationship was identified between acetoacetate and HbA1c levels (*β* = 0.0002; *p* = 0.0154). *GU174097_g* reduced ketone body levels, thus decreasing HbA1c levels and the risk of T2D. Taken together, our findings indicate that *GU174097_g* may lower the risk of T2D by reducing ketone body levels.

## Diabetes: a little help from the microbiome

A microbe that may help protect against type II diabetes has been detected by examining extracellular vesicles (EVs), tiny membrane-wrapped packages secreted by human cells and by the bacteria making up the microbiome. Examining EVs allows researchers to sample microbial populations other than the intensively studied intestinal microbiome. Sungho Won, Seoul National University, and Geum-Sook Hwang, Korea Basic Science Institute, Seoul, and coworkers studied the microbial EVs in urine samples collected from South Korean subjects over four years. They identified a previously unclassified bacterial species in the family Lachnospiraceae that was associated with lower risk of developing type II diabetes. Further investigation showed that these bacteria may break down ketone bodies, metabolic byproducts that signal disrupted sugar metabolism leading to diabetes. These results contribute to understanding how the microbiome contributes to metabolic health and disease.

## Introduction

Recent studies have revealed that the intestinal microbiota plays essential roles in host energy homeostasis, body adiposity, blood sugar control, insulin sensitivity, hormone secretion, and the pathogenesis of metabolic diseases, such as type 2 diabetes (T2D) and obesity^1–3^. However, most of these studies analyzed stool samples and therefore obtained limited information relative to insights from direct sampling of the intestinal mucosa, which is not possible in most cases. In addition, the composition of microbial communities in stool samples is greatly affected by the specific compartment in which they reside, such as the mucous membrane^4^. Microbial communities also differ based on their source, ranging from the intestines, skin, and airways, which are frequently studied, to urine and blood, which are generally sterile environments^5^. Therefore, it is important not only to understand the role of the intestinal microbiota but also to consider the function and combined contribution of the all microbiota throughout the body.

Extracellular vesicles (EVs) have been recently suggested to function as the main messengers between intestinal microorganisms and the host. EVs travel long distances within and between body tissues^6^ and have been used as biomarkers of atopic dermatitis, alcoholic hepatitis, and asthma^7–10^. Microbiota-derived EVs can enter the circulatory system through the intestinal barrier. They are suspected to play a key role in the development of insulin resistance, potentially providing important clues into the pathogenesis of T2D. For example, EVs derived from *Pseudomonas panacis* are present in the stool samples of high-fat diet-fed mice. They can infiltrated the gut barrier and block the insulin pathway in skeletal muscle and adipose tissue, inducing the development of insulin resistance and glucose intolerance^11^. However, microbiota-derived EVs are highly variable, as they are modulated by different factors, such as age and sex. Therefore, caution should be exercised when inferring causal relationships based on the statistical analysis of microbiota-derived data. Furthermore, longitudinal microbiota studies may allow for stronger inferences than cross-sectional studies^12^ and may allow for the detection of microorganisms related to the progression of T2D in healthy subjects. However, existing studies have been predominantly cross-sectional in nature and are based on correlation analyses. As a result, these studies are unable to comprehensively provide an understanding of the exact roles of the intestinal microbiota and EVs in metabolic disease development.

Therefore, in the present study, we investigated the prospective Korean Association REsource project (KARE) cohort^13^. By tracking changes in microbiota-derived EVs in urine samples from Korean adults collected three times over four years, we explored the potential associations between microorganisms and T2D progression. Furthermore, using genomic and metabolite data from the KARE cohort, we conducted a multiomics analysis to investigate the specific role of microorganisms potentially involved in T2D pathogenesis. We expect our findings to provide information regarding how microbes, the substances they produce, and their byproducts interact with the human body and affect metabolic disease development. In addition, we evaluated causal relationships among microbial organisms, ketone bodies, and clinical measurements, with the aim of further elucidating the relationship between T2D and the microbiota.

## Materials and methods

### Cohort and study design

**Fig. 1:**
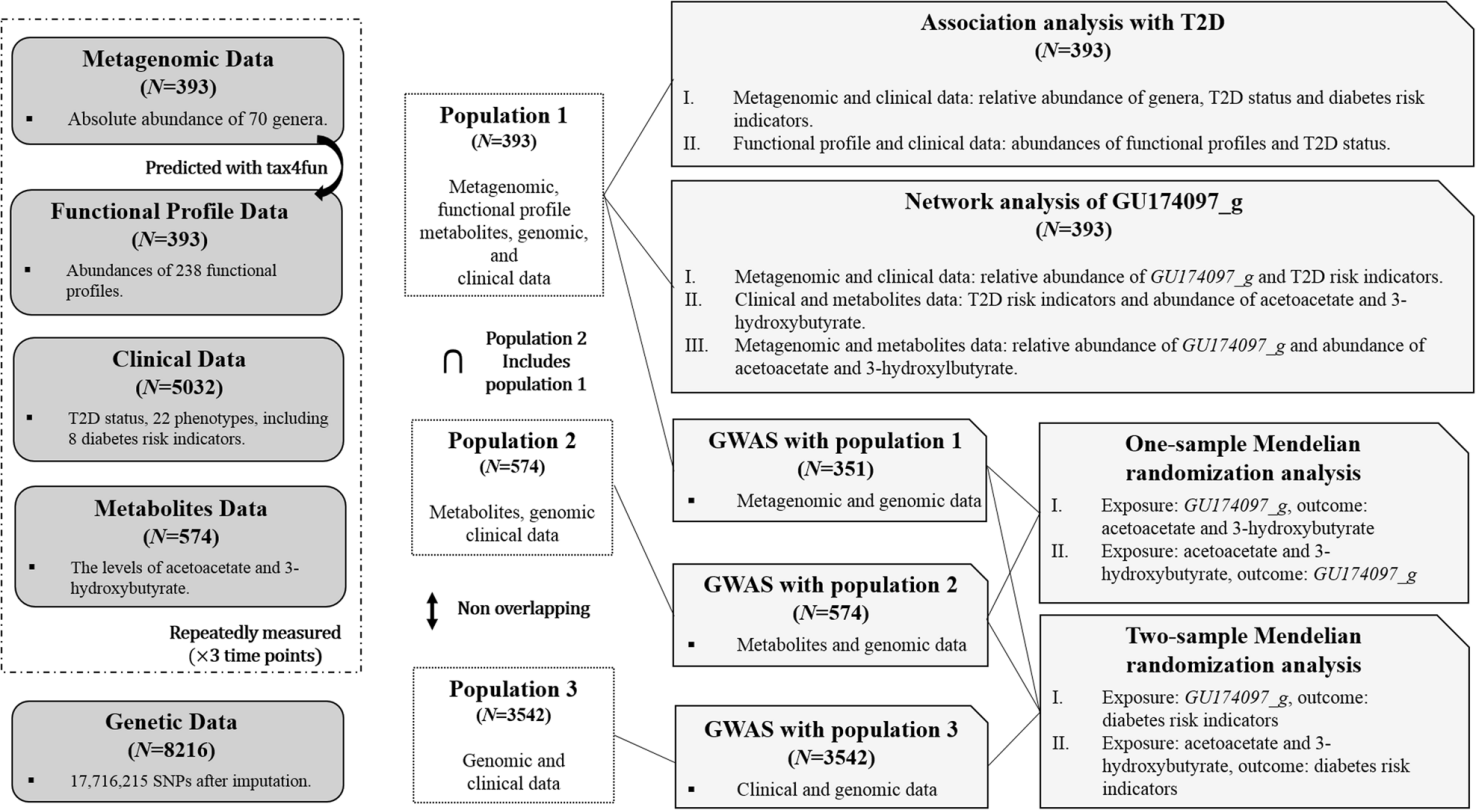
Summary chart of data analysis. Datasets and analyses employed in the current study are described. Sample sizes for each analysis are presented.

The KARE cohort is a prospective study cohort involving subjects from the rural community of Ansung and the urban community of Ansan in South Korea. The KARE project began in 2001 as part of the Korean Genome Epidemiology Study^14^. We used data from urine samples taken from subjects in 2013, 2015, and 2017, which we refer to as phases 1, 2, and 3 in this study. After collection, the urine was stored at –80 °C. For the 1,891 subjects whose urine samples were available, age, sex, and body mass index (BMI) were matched via 2:1:1 propensity score matching. As a result, a healthy group (healthy in all phases, *N* = 328), a T2D-at-risk group (T2D-at-risk in all phases, *N* = 164), and a T2D group (T2D in any of the three phases, *N* = 164) were selected. From the remaining unmatched subjects, 35 T2D subjects were also included. Consequently, 691 subjects were finally included, and their 2,072 urine samples were subjected to microbiota analysis. Metagenomic, metabolite, clinical, and genomic data were subjected to comprehensive analyses (Fig. 1).

### Operational definition of T2D and related phenotypes

Study participants were categorized into control individuals, T2D-at-risk patients, and T2D patients. T2D and T2D-at-risk patients were diagnosed on the basis of the American Diabetes Association criteria, which are provided in Supplementary Table 1. T2D status was then stratified into *T2D-at-risk/T2D* (0 for healthy; 1 for T2D-at-risk and T2D) and *binary_T2D* (0 for healthy and T2D-at-risk; 1 for T2D). In addition, we considered other T2D-related indicators, such as BMI, HbA1c levels, fasting glucose and insulin levels, 60- and 120-min plasma glucose levels, and insulin levels after a 75 g oral glucose tolerance test in our analysis. Age, the levels of total cholesterol, high-density lipoprotein (HDL) cholesterol, triglycerides, kidney- and liver-related disease indicators (blood urea nitrogen (BUN), creatinine, aspartate aminotransferase (AST), and alanine aminotransferase (ALT) C-reactive protein (CRP), white blood cell (WBC) count, red blood cell (RBC) count, hemoglobin, hematocrit, and platelet count) were also collected. The homeostatic model assessment for insulin resistance (HOMA-IR) was calculated using fasting glucose and fasting insulin levels^15^. Descriptive statistics for all variables were generated using Rex software (RexSoft Inc., Seoul, Korea) (Supplementary Table 2)^16^.

### EV isolation and DNA extraction

For EV isolation, urine samples were subjected to differential centrifugation at 10,000 × g and 4 °C for 10 min using a microcentrifuge (Labogene 1730R; Bio-Medical Science, Seoul, Korea)^17^. To remove bacteria, foreign particles, and waste, the supernatant was filtered through a 0.22-µm filter (Inchpor2 Syringe Filter; Inchemtec, Seoul, Korea). The isolated EVs were boiled at 100 °C for 40 min and centrifuged at 18,214 × g and 4 °C for 30 min to eliminate floating particles and impurities. The supernatant was collected and subjected to DNA extraction using a PowerSoil^®^ DNA Isolation Kit (MO BIO Laboratories, Carlsbad, CA, USA) according to the manufacturer’s protocol. DNA was quantified using the QIAxpert system (Qiagen, Hilden, Germany).

### 16 S rRNA sequence data processing

Paired-end sequencing of the V3-V4 region of the bacterial 16 S rRNA gene was conducted at MD Health care (Seoul, Korea) with the MiSeq Reagent Kit v3 (600 cycles, Illumina, San Diego, CA, USA) using the widely used primers 16S_V3_F (5′-TCGTCGGCAGCGTCAGATGTGTATAAGAGACA-GCCTACGGGNGGCWGCAG-3′) and 16S_V4_R (5′-GTCTCGTGGGCTCGGAGATGTGTATA-AGAGACAGGACTACHVGGGTATCTAATCC-3′). Adaptor sequences were detected and removed using CUTADAPT software (https://cutadapt.readthedocs.io) with a minimum overlap of 11, a maximum error rate of 10%, and a minimum length of 10^18^. Sequences were merged using CASPER (http://best.snu.ac.kr/casper) with a mismatch ratio of 0.27 and filtered based on the Phred (Q) score, resulting in sequences of 350–550 bp in length^19,20^. After the merged sequences were dereplicated, chimeric sequences were detected and removed using VSEARCH (https://github.com/torognes/vsearch) and the Silva Gold reference database for chimeras^21^. Open-reference operational taxonomic unit (OTU) picking was conducted based on the EzTaxon database using UCLUST (http://www.drive5.com/usearch)^22,23^. For each OTU, we calculated its proportion among all OTUs and determined the mean value across all subjects. If the resulting value was <0.001, the OTU was excluded^24^. Among the 691 subjects, those with a read count <3000 or whose genomic data were not available in any phase were excluded. As a result, 1179 samples from 393 subjects, including 70 genera, were used for subsequent analyses.

### Prediction of functional profiles from 16 S rRNA metagenomic data

The functional potential of microbial communities can be predicted from their phylogeny. Tax4fun uses evolutionary modeling to predict metagenomes based on 16 S data from the SILVA reference genome database. The SILVA-based 16 S rRNA profile was used to estimate a taxonomic profile of prokaryotic Kyoto Encyclopedia of Genes and Genomes (KEGG) organisms. The estimated abundances of KEGG organisms were normalized using the 16 S rRNA copy number obtained from the National Center for Biotechnology Information (NCBI) genome annotations. Finally, the normalized taxonomic abundances were used to linearly combine the precomputed functional profiles of KEGG organisms to predict the functional profile of the microbial community^25^. Similar to the analysis of OTUs, we calculated the mean of the relative proportions across all subjects for each functional profile. If the resulting value was <0.001, the functional profile was excluded from the analysis. As a result, 238 functional profiles were retained for analysis.

### Metabolite analysis of ketone bodies

Serum metabolites were analyzed using the Agilent 1290 Infinity LC and Agilent 6490 Triple Quadrupole MS systems (Agilent Technologies, Palo Alto, CA, USA). The levels of acetoacetate and 3-hydroxybutyrate from subjects included in the metagenomics dataset were determined in the multiple reaction monitoring mode. A batch normalizer was used to correct for possible batch effects^26^.

### Analysis of bacterial composition and microbial variance

We calculated alpha- and beta-diversity indices using R (v3.6.2) after read number normalization with the Rarefy function in the R package GUniFrac (v1.1). The R package Fossil (v0.4.0) was used to obtain Chao1 and ACE diversity indices. The Shannon index and Simpson’s diversity index were calculated using the Vegan package in R (v2.5.6). Taxonomy-based ring charts were created using the Krona tool^27^. PERMANOVA is a nonparametric multivariate analysis of variance test based on pairwise distances^28^. The R package pldist was used to obtain the microbial variance for individuals in repeated measurements of microbial profiles. pldist summarizes within-individual shifts in the microbiome composition and compares these across individuals. pldist also calculates dissimilarities based on a novel transformation of relative abundances, which are then extended to more than two time points. They are then incorporated into a chosen beta-diversity, which, in our case, was Bray–Curtis dissimilarity. PERMANOVA was performed for biochemistry-related KARE phenotypes using the adonis function in R. PERMANOVA can be applied to the cross-sectional data, and thus, the phenotypes were averaged for phases 1, 2, and 3.

### Statistical analysis of the effect of the microbiome on T2D and diabetes risk indicators

For each taxon and functional profile, a generalized linear mixed model (LMM) with the logit link function was used to find associations with *binary_T2D* and *T2D-at-risk/T2D*, whereas an LMM was used for log-transformed diabetes risk indicators. A random effect with a compound symmetry structure for each time point was incorporated to adjust the similarity of T2D status for the same subject at different time points, and the sandwich estimator was used to find a robust estimate against the misspecified covariance matrix. To accommodate the multiple testing problem, *p* values were adjusted for the false discovery rate (FDR) using the Benjamini–Hochberg method^29^.

### Network analysis of a T2D-related taxon based on multiomics data

To assess overall associations using repeatedly measured multiomics data, we first modeled an LMM using log-transformed diabetes risk indicators as response variables and age in phase 1 as well as sex as explanatory variables with a compound symmetry structure for its covariance. We modeled an LMM with a T2D-related taxon as the response variable with the same covariates and covariance structure. For each combination of diabetes risk indicators and a T2D-related taxon, two different sets of residuals were obtained, and Spearman correlations between the residuals were calculated. Similarly, the association between a chosen microbial marker and the levels of ketone bodies was analyzed.

Network analysis was conducted to calculate simple correlations among diabetes risk indicators, a chosen taxon, and ketone bodies. Edge width was calculated as –log_10_ of the *p* value. The network was visualized using the R package visNetwork (v2.0.8).

### Genotyping, imputation, and quality control

Quality control and genotype imputation were performed according to the standard quality control and imputation protocols for the genotypes of 8842 KARE cohort participants^30^. After quality control, 8216 subjects with 17,716,215 single-nucleotide polymorphisms (SNPs) were included in the analysis. In total, the data of 351 subjects with a read count <3,000 and nonmissing T2D status for all phases were used for a genome-wide association study (GWAS) of metagenomic data. A total of 574 subjects who had no missing metabolite levels and T2D status for all three phases were selected for a GWAS of metabolite levels. Among the subjects not included in the metabolite or metagenome GWAS, 3542 subjects had KARE phenotypes for the three phases and were thus included in a GWAS of KARE phenotypes. We excluded subjects in the metabolite or metagenome GWAS for the purposes of a two-sample Mendelian randomization (MR) study. Details are provided in Supplementary Fig. 1, and all the associated SNPs from each GWAS are listed in Supplementary Table 3.

### MR analysis

MR uses genetic variants that are not associated with conventional confounders of observational studies and is therefore considered analogous to randomized controlled trials^31^. Randomly selected alleles are transmitted from parents, and genotypes can be assumed to be independent, with many potential confounders. This randomization produces unbiased estimates for the associations between the main exposures and outcomes. Thus, genetic variants associated with the main exposure were used as instrumental variables. There are two types of MR, namely, two-sample MR and one-sample MR. The former uses two independent datasets with nonoverlapping samples for the association of SNP exposure and SNP outcome (as opposed to one-sample MR) It is less likely to lead to inflated type 1 error rates and false-positive findings when compared to one-sample MR. Two-sample MR was conducted to identify the effect of a microbial taxon or each ketone body on KARE phenotypes by using no overlapping samples. One-sample MR was conducted to estimate the effect of a chosen taxon on each ketone body.

For one-sample MR, we conducted two-stage least-squares regression. The first stage consisted of a regression for SNP exposure, and the second stage consisted of a regression for the outcome of interest on the fitted values from the first-stage regression. The estimator of the coefficient for first-stage fitted values in the second-stage model is the causal estimate^32,33^. F-statistics from the first-stage regression were examined to avoid weak instrument bias^34^. The Durbin-Wu-Hausman (DWH) test for endogeneity^35^ was used to evaluate whether there is any evidence that the causal estimate differs from the ordinary least square estimate of exposure and outcome. For two-sample MR, the average *F*-statistic was used to avoid weak instrument bias. The inverse-variance-weighted (IVW) method, Cochran’s *Q* test, and MR-PRESSO global test were used to confirm the heterogeneity assumption, and *I*^2^ was used for the no measurement error (NOME) assumption. To enhance the validity of MR analysis, we considered the extensive range of existing MR methods, including IVW, MR-egger, MR-egger with SIMEX correction, median-weighted method, and MR-PRESSO, and selected the recommended MR method based on the violations of MR assumptions^36^.

## Results

### Longitudinal changes in the urine microbial composition over four years

**Fig. 2:**
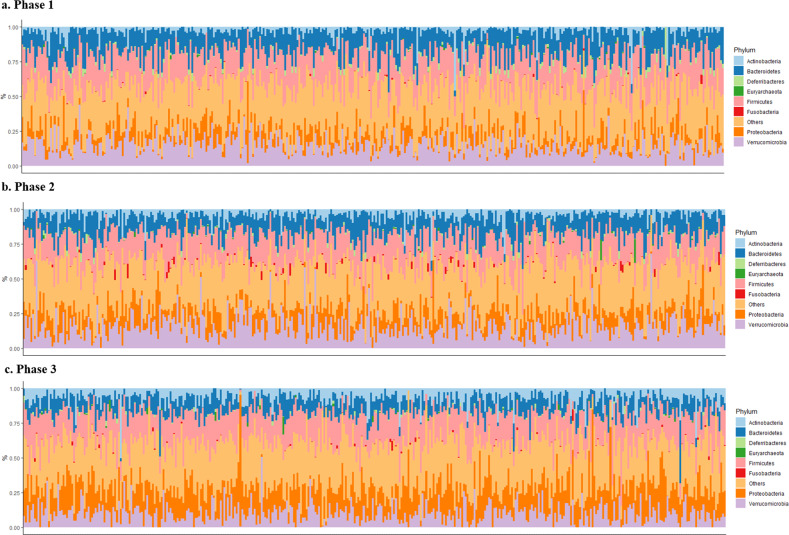
Taxonomic composition at the phylum level for phases 1, 2, and 3. Profile plots for phases 1 (**a**), 2 (**b**), and 3 **c** showing the relative abundances of bacterial taxa at the phylum level for each individual are presented.

The alpha-diversity of the urine microbiome decreased during the follow-up period, which may have been an effect of aging (Supplementary Fig. 2). A nonmetric multidimensional scaling plot based on beta diversity also revealed a gradual change in microbiota composition with age (Supplementary Fig. 3). The overall microbiome composition at the phylum and genus levels is presented in Fig. 2 and Supplementary Fig. 4, respectively. *Verrucomicrobia*, *Bacteroidetes*, and *Firmicutes* were the predominant phyla, whereas *Akkermansia* and *Bacteroides* were the predominant genera.

### T2D and other clinical traits explained by microbial variance

We investigated the associations between various clinical phenotypes and microbial compositions using PERMANOVA (Supplementary Fig. 5). HbA1c, WBC, hematocrit, *binary_T2D*, and age in phase 1 significantly explained changes in microbial composition during the follow-up period (*p* = 0.0061, 0.0107, 0.0110, 0.0409, and 0.0290, respectively; FDR-adjusted *p* = 0.1027, 0.1027, 0.1027, 0.2290, and 0.2030, respectively). HbA1c and *binary_T2D* partially explained the variance in microbial changes over the 4 years, indicating that the longitudinal change in microbiome composition may be more closely associated with T2D-related phenotypes than with other clinical traits.

### Taxa and functional profiles associated with T2D and diabetes risk indicators

**Fig. 3:**
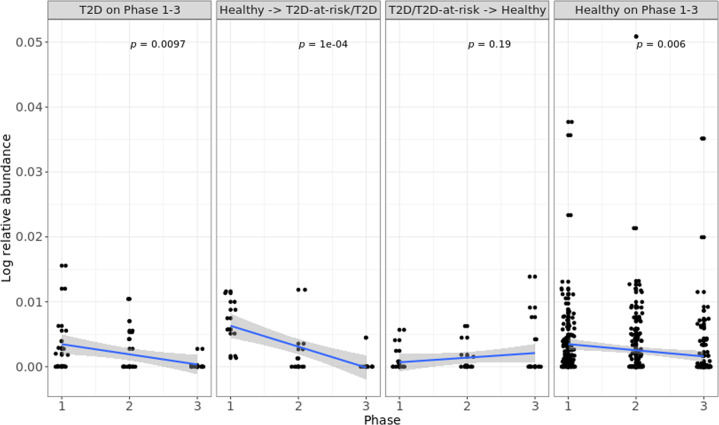
Relative proportions of GU174097_g in different type 2 diabetes (T2D) groups. The mean relative proportions of *GU174097_g* are provided for the *Healthy in Phases 1-3*, *T2D in Phases 1-3*, *Healthy to T2D-at-risk/T2D*, and *T2D-at-risk/T2D to Healthy* groups and are compared according to the T2D status. The *Healthy in Phases 1-3* group included subjects who were healthy in phases 1, 2, and 3. The *T2D in Phases 1-3* group included subjects who had T2D in phases 1, 2, and 3. The *Healthy to T2D-at-risk/T2D* group included subjects who were healthy in phase 1 and became T2D patients or T2D-at-risk in phase 3. The *T2D-at-risk/T2D to Healthy* group included subjects who were T2D-at-risk/T2D in phase 1 and healthy in phase 3. The *p* values were calculated based on simple linear regression using the log relative proportion of *GU174097_g* as a response variable and phase variable. The relative proportion of *GU174097_g* was log-transformed after adding one to avoid zero values. The phase variable is coded by 1, 2, and 3 for phases 1, 2, and 3, respectively.

In an association analysis of 70 genera with *binary_T2D* and *T2D-at-risk/T2D* phenotypes, *GU174097_g*, an unclassified *Lachnospiraceae*, was found to exhibit a significant association with these phenotypes and was more abundant in healthy subjects than in diabetic or prediabetic patients (Table 1). We divided the samples into four groups. The *Healthy in Phases 1-3* group included subjects who were healthy in phases 1, 2, and 3. The *T2D in Phases 1-3* group consisted of subjects who had T2D in phases 1, 2, and 3. The *Healthy to T2D-at-risk/T2D* group included subjects who were healthy in phase 1 and became T2D patients or T2D-at-risk in phase 3. The *T2D-at-risk/T2D to Healthy* group included subjects who were T2D-at-risk/T2D in phase 1 and healthy in phase 3. The relative abundance of *GU174097_g* in subjects who were healthy at baseline but changed to the *T2D-at-risk/T2D* group at phase 2 or 3 decreased with the development of T2D (*p* = 0.0001). Conversely, its relative abundance in the *T2D-at-risk/T2D to Healthy* group exhibited no tendency to decrease (*p* = 0.19) (Fig. 3). Supplementary Fig. 6 shows the profiles of *GU174097_g* for randomly selected subjects. The relative abundance of *GU174097_g* in subjects who were healthy at baseline but changed *to T2D-at-risk/T2D* at phase 2 or 3 tended to decrease. Most T2D patients had small relative abundances of *GU174097_g* at baseline. In summary, *GU174097_g* was clearly associated with the progression of diabetes over time, and this association was not simply based on diabetic or nondiabetic status.

**Table 1:** Analysis of the associations between type 2 diabetes (T2D) and bacterial genera.

Phenotype	Genus	Estimate	Std Err	DF	*p value*	FDR
*T2D-at-risk/T2D*	*GU174097_g*	−189.13	46.63	735	0.00006	0.00393
*Binary_T2D*	*JN713389_g*	−13.07	5.31	735	0.01411	0.38195
*Binary_T2D*	*Akkermansia*	−3.49	1.43	735	0.01489	0.38195
*Binary_T2D*	*Dialister*	−86.44	37.49	735	0.02140	0.38195
*Binary_T2D*	*Ruminococcus_g2*	−25.38	11.70	735	0.03039	0.38195
*Binary_T2D*	*KE159538_g*	−48.29	22.95	735	0.03568	0.38195
*Binary_T2D*	*Bifidobacterium*	6.71	3.21	735	0.03669	0.38195
*Binary_T2D*	*Eubacterium_g8*	−71.10	34.46	735	0.03944	0.38195
*Binary_T2D*	*Megamonas*	−65.20	32.53	735	0.04538	0.38195
*Binary_T2D*	*Pseudomonas*	7.74	3.91	735	0.04842	0.38195

Associations between genera and *T2D-at-risk/T2D* and *Binary_T2D* were tested, and significant associations at a significance level of 0.05 are summarized.

To investigate the T2D-associated microbial functional profiles, 238 functional profiles were evaluated. The significant associations at an FDR-adjusted significance of 0.1 are presented in Supplementary Table 4. The *T2D-at-risk/T2D* phenotype was related to the cationic antimicrobial peptide. Furthermore, the biosynthesis of fatty acids, coenzyme A (CoA), and secondary metabolites as well as oxidative phosphorylation were significantly associated with the *Binary_T2D* phenotype at an FDR-adjusted significance of 0.1.

Next, we investigated the associations between the log-transformed diabetes risk indicators and genera, and significant associations at an FDR-adjusted significance of 0.1 were identified. Twelve, four, and 20 genera were significantly associated with HbA1c, glucose, and insulin levels, respectively. In particular, *Hafnia* was associated with HbA1c and 60- and 120-min insulin levels, whereas *AB185816_g* and *Akkermansia* were associated with HbA1c, fasting glucose, and 60-min insulin levels (Supplementary Table 5).

### Associations between T2D-related unclassified *Lachnospiraceae* and diabetes risk indicators and ketone bodies

To confirm the association between *GU174097_g* and T2D, we performed extensive validation analysis using clinical and metabolite data. We analyzed the association between *GU174097_g* and diabetes risk indicators (Table 2). Among all glucose- and insulin-related variables, *GU174097_g* was significantly and positively associated with the 60-min insulin level.

**Table 2:** Analysis of the associations between diabetes risk indicators and *GU174097_g*.

Phenotype	rssho	*p* value	FDR
Ins60	0.0950	0.0018	0.0026
HbA1c	−0.0434	0.1548	0.1872
Glu0	0.0306	0.3165	0.3647
Ins0	−0.0301	0.3236	0.3721
HOMA-IR	−0.0270	0.3756	0.4255
Glu60	−0.0229	0.4536	0.5045
Ins120	0.0177	0.5622	0.6096
Glu120	0.0072	0.8138	0.8424
BMI	−0.0070	0.8186	0.8469

Associations between diabetes risk indicators and *GU174097_g* were tested and are presented.

Thereafter, we analyzed the potential associations between ketone bodies and the T2D-related taxon, since ketone bodies have been suggested as markers of disrupted glucose metabolism in prediabetic patients^37^. The ketone bodies 3-hydroxybutyrate and acetoacetate exhibited significant negative correlations with *GU174097_g* (*r* = –0.0829 and –0.0938, respectively; *p* = 0.0069 and 0.0022, respectively) (Table 3). Supplementary Fig. 7 shows the tendency of high acetoacetate and 3-hydroxybutyrate concentrations coinciding with the low abundance of *GU174097_g*. The ion abundances of acetoacetate and 3-hydroxybutyrate did not rise beyond 2000 and 10000, respectively, when the relative abundance of *GU174097_g* was high.

**Table 3:** Analysis of the associations between *GU174097_g* and ketone bodies.

Phenotype	rho	*p* value
Acetoacetate	−0.0938	0.0022
3-hydroxybutyrate	−0.0829	0.0069

Associations between the ketone bodies acetoacetate and 3-hydroxybutyrate and *GU174097_g* were tested and are presented.

Finally, we established an association network for diabetes risk indicators and ketone bodies, as the same observed correlations can imply completely different biological processes. For example, if high levels of glucose or HbA1c tend to appear in parallel to high levels of insulin, insulin resistance may be present. However, if high levels of glucose or HbA1c are observed in parallel to low levels of insulin, insulin secretion may have suppressed glucose or HbA1c levels. Network analysis indicated strong associations among the diabetes risk indicators (Supplementary Fig. 8). In particular, the 60-min insulin level exhibited a strong negative correlation with HbA1c levels, suggesting that the former can decrease the latter. Ketone bodies exhibited negative correlations with fasting insulin and 60-min insulin levels and positive correlations with 60- and 120-min glucose levels.

### Causal relationship between the T2D-related taxon and ketone bodies and the diabetes risk indicators

One-sample MR did not reveal any significant causal relationship between *GU174097_g* and ketone bodies and vice versa (Supplementary Table 6). To verify whether a causal relationship existed between the abundance of *GU174097_g* or the levels of ketone bodies and diabetes risk indicators, two-sample MR analysis was performed. Extensive assumption checks were conducted to enhance the validity of the two-sample MR analysis (Supplementary Table 7). No weak instrument bias was observed (*F*-statistic >10). However, NOME assumptions were violated for all tests because *GU174097_g*, 3-hydroxybutyrate, and acetoacetate had seven, eight, and five SNPs as their instrument variables, respectively, and these values were not sufficiently large for *I*^2^ > 90. In this case, if heterogeneity exists, MR–Egger (SIMEX) is recommended; otherwise, IVW is recommended. As the InSIDE assumption cannot be statistically tested^38^, the weighted median method—a robust approach used in cases of InSIDE assumption violation—has to be considered with each recommended method^36^. Therefore, MR–Egger (SIMEX) was used to estimate the causal effect of 3-hydroxybutyrate on 60-min insulin as well as that of acetoacetate on HbA1c levels. The IVW method was used to estimate all other causal effects. To determine the causal effect of 3-hydroxybutyrate on 60-min insulin, rs2259835 was detected as an outlier via MR-PRESSO at a significance level of 0.05 (Supplementary Table 8). Thus, rs2259835 had to be removed to prevent potential horizontal pleiotropy. The result of MR-PRESSO is shown in Supplementary Table 9 and shows the estimates without outliers. The effect of acetoacetate on the HbA1c level was the only significant effect at an FDR-adjusted significance of 0.05, indicating that acetoacetate increases HbA1c levels (Supplementary Table 9). The results obtained using the weighted median method corroborated this significant association (*p* = 0.0475).

## Discussion

Recent microbiome studies have shown that T2D is associated with gut dysbiosis^39–41^ that can result in altered intestinal barrier function and a dysregulation of host metabolic and signaling pathways^42^. Intestinal bacteria can promote insulin resistance by triggering inflammation via polysaccharides, which are components of the gram-negative bacterial cell wall^43^. Furthermore, microbiota-derived EVs are expected to affect insulin resistance and provide a more in-depth understanding of T2D pathogenesis^11^. Various bacterial metabolites, such as short-chain fatty acids (SCFAs), can modulate the function of various signaling pathways implicated in human health and can protect against insulin resistance^43,44^.

The human microbiota is highly variable, and this variability is determined by various external factors, such as diet, exercise, mobility, medication, and microbial cooccurrence patterns. Many of these external factors also determine the risk of metabolic disease and are age-related^45^; that is, the intestinal microbiota and host phenotype are substantially altered with aging. Furthermore, the effect of the intestinal microbiota on the host phenotype is also dependent on the age of the host. The estimation of within-subject covariate effects represents a robust approach against between-subject confounders, and longitudinally measured microbiome data enable characterization of the effects of the microbiota on host disease risk. As most existing studies have been cross-sectional in nature, the validity and interpretation of their results are limited. In turn, longitudinal studies are needed to comprehensively investigate the association between the human microbiome and diseases, including T2D.

Our longitudinal study revealed that a low abundance of *GU174097_g* is a risk factor for T2D development. *GU174097_g* has not been cultured to date. Multiomics data, including host genomic data, T2D-related metabolites, clinical information, and predicted functional metagenomic profiles, were utilized to extensively validate our results via causality analysis. *GU174097_*g is a member of the family *Lachnospiraceae*, and an association between *Lachnospiraceae* and T2D risk has been reported in several previous studies^46,47^. SCFA pathways, including the propanediol and acrylate signaling pathways, play important roles in mediating the effects of *Lachnospiraceae* on T2D^45^. Additionally, SCFA-producing bacteria affect epigenetic regulation in T2D patients and reduce the risk of developing T2D^44,48^. We found that *GU174097_g* is positively correlated with the 60-min insulin level, and in turn, it is negatively correlated with HbA1c levels. This indicates that *GU174097_g* reduces HbA1c levels and, thus, the risk of developing T2D by stimulating insulin secretion.

Next, we aimed to elucidate how *GU174097_g* affects T2D through the regulation of 60-min insulin and HbA1c. Multiple mechanisms may underlie these associations, including the effects of various microbiota-derived metabolites, including SCFAs, as previously suggested. In addition, ketone bodies have been reported not only as indicators of diabetic hyperglycemia but also as markers of disturbed glucose metabolism in the prediabetic state^37,48^. Furthermore, fatty acid metabolism, CoA synthesis, and oxidative phosphorylation, all of which are involved in ketogenesis or ketolysis, have been associated with T2D^49^. In our study, the ketone bodies 3-hydroxybutyrate and acetoacetate were negatively correlated with *GU174097_g* but positively correlated with the 60- and 120-min glucose levels. MR analysis was employed to investigate the effects of *GU174097_g* and ketone bodies on diabetes risk indicators. Although no causal relationship was observed between *GU174097_g* and ketone bodies or other clinical variables, acetoacetate was found to be causally related to an increased HbA1c level. HbA1c level is a major biomarker of T2D and explains the microbial beta-diversity. Furthermore, *GU174097_g* was negatively correlated with acetoacetate. Therefore, our study not only confirmed the importance of ketone bodies in T2D pathogenesis but also suggests an underlying mechanism for the association between *GU174097_g* and T2D development.

Previous studies have reported that gut microbe–derived EVs can infiltrate the circulatory system through the gut barrier^11,50^. Furthermore, microbe-derived EVs in urine can reflect the lung and gut microbiota of children with asthma^51^. Interestingly, T2D increases the co-occurrence of the same OTUs within the gut microbiome and microbe-derived EVs in urine samples^52^, which indicates that these EVs may reflect the gut microbiota composition. *Coprococcus*, a member of the *Lachnospiraceae* family, is one of the major butyrate-producing bacteria. It is known to utilize metabolic intermediates essential for the synthesis of ketone bodies, such as acetoacetyl-CoA, 3-hydroxybutyryl-CoA, and crotonyl-CoA26, as energy sources to produce the SCFA butyrate. SCFAs are considered beneficial for health and are considered to protect against T2D^53^. Thus, we hypothesize that *GU174097_g* consumes acetoacetate to produce SCFAs. These SCFAS can promote insulin secretion and decrease HbA1c levels, leading to a decreased risk of T2D.

Our study had several limitations. First, as it was based on the metagenomic profiles of EVs, the microbial compositions observed can differ from, and need to be further compared with, those of the intestinal microbiota. Second, as the genus-level taxonomy of *GU174097_g* is unknown, ecological and biological information on this species is limited. Third, published summary statistics of microbial GWAS are limited, and the sample size in the current microbial GWAS was small. Therefore, the number of SNPs used as instrumental variables in our MR analysis was also suboptimal. Future studies should include a large sample size to identify more associated SNPs and increase the power of MR analysis. Therefore, the mechanisms underlying T2D pathogenesis could be further identified and characterized. Fourth, even though extensive methods were used to validate assumptions in our MR analysis and enhance the validity of causal analysis, the MR results were not easy to interpret. Ketone bodies and diabetes risk indicators were highly correlated and interacted with each other. Additional in vivo and in vitro experiments may clarify the associations identified herein.

Our study revealed that *GU174097_g*, an unclassified *Lachnospiraceae*, is associated with T2D and ketone bodies. Furthermore, we found a potential causal relationship between ketone body acetoacetate and HbA1c levels. Our findings indicate that *GU174097_g* may lower the risk of developing T2D via the reduction in ketone body levels. Although the mechanisms by which *GU174097_g* and ketone bodies affect T2D development have not been elucidated, further large-scale longitudinal studies as well as in vivo and in vitro experiments could contribute to unraveling these mechanisms.

## Supplementary information

Supplementary information

## Data Availability

Raw datasets generated during the current study are available in the NCBI Sequence Read Archive (BioProject id PRJNA716550; SRA accession id SAMN18437890-SAMN18438579, SAMN18443936-SAMN18444626, SAMN18446963-SAMN18447653).
