# Supplementary material for: Role of an unclassified Lachnospiraceae in the pathogenesis of type 2 diabetes: a longitudinal study of the urine microbiome and metabolites
Source: Exp Mol Med. 2022 Aug 5;54(8):1125–32. doi: 10.1038/s12276-022-00816-x (PMC9440228; doi:10.1038/s12276-022-00816-x)

Supplementary information

Role of an unclassified Lachnospiraceae in the pathogenesis of type 2 diabetes: A longitudinal study of the urine microbiome and metabolites

Supplementary Table 1. Criteria used for the diagnosis of type 2 diabetes (T2D) and T2D-at-risk patients

Diagnosis Condition	Fasting glucose (mg/dL)	120 min glucose (mg/dL)	HbA1c (%)	Diabetic medicine taken
Without Diabetes	<100	<140	<5.7	No
T2D-at-risk (Prediabetes)	100–125	140–199	5.7–6.4	No
T2D (Diabetes)	≥126	≥200	≥6.5	Yes

The criteria are provided by the American Diabetes Association.

Supplementary Table 2. Basic demographic and clinical characteristics of KARE cohort subjects

Variable	Category	Statistics	Phase 1 (*N*=393)	Phase 2 (*N*=393)	Phase 3 (*N*=393)	Total (*N*=1179)
Sex						
	Male	n(%)	192(48.85%)	192(48.85%)	192(48.85%)	576(48.85%)
	Female	n(%)	201(51.15%)	201(51.15%)	201(51.15%)	603(51.15%)
	Total	n(%)	393(100.00%)	393(100.00%)	393(100.00%)	1179(100.00%)
Age						
		n	393	393	393	1179
		mean ± Std	57.23 ± 5.88	59.20 ± 5.91	61.29 ± 5.93	59.24 ± 6.13
		Q1, Q3	53.00, 60.00	55.00, 62.00	57.00, 64.00	55.00, 63.00
		min ~ max	49.00 – 77.00	51.00 – 79.00	53.00 – 81.00	49.00 – 81.00
Hemoglobin						
		n	393	393	393	1179
		mean ± Std	5.55 ± 0.42	5.55 ± 0.45	5.67 ± 0.60	5.59 ± 0.50
		Q1, Q3	5.30, 5.80	5.30, 5.80	5.30, 5.90	5.30, 5.80
		min ~ max	4.10 – 7.40	4.20 – 7.90	4.30 – 11.10	4.10 – 11.10
Glu0						
		n	393	393	393	1179
		mean ± Std	92.52 ± 9.47	92.06 ± 11.58	94.80 ± 14.44	93.12 ± 12.05
		Q1, Q3	86.00, 97.00	85.00, 96.00	87.00, 99.00	86.00, 98.00
		min ~ max	71.00 – 141.00	62.00 – 159.00	71.00 – 224.00	62.00 – 224.00
Glu60						
		n	372	364	344	1080
		mean ± Std	161.60 ± 46.01	164.50 ± 49.51	172.60 ± 48.88	166.08 ± 48.30
		Q1, Q3	127.00, 194.00	127.00, 199.00	140.00, 204.00	130.00, 200.00
		min ~ max	50.00 – 304.00	66.00 – 373.00	54.00 – 423.00	50.00 – 423.00
Glu120						
		n	372	364	344	1080
		mean ± Std	136.10 ± 40.59	141.66 ± 43.85	144.25 ± 53.05	140.57 ± 46.04
		Q1, Q3	105.75, 158.25	112.00, 166.00	106.75, 173.25	107.00, 166.00
		min ~ max	47.00 – 287.00	58.00 – 331.00	61.00 – 447.00	47.00 – 447.00

(Continued)

Supplementary Table 2. Continued

Variable	Category	Statistics	Phase 1 (N=393)	Phase 2 (N=393)	Phase 3 (N=393)	Total (N=1179)
BUN						
		n	393	393	393	1179
		mean ± Std	15.37 ± 3.90	15.71 ± 3.93	15.90 ± 3.83	15.66 ± 3.89
		Q1, Q3	12.60, 17.60	12.90, 18.20	12.90, 18.10	12.90, 18.00
		min ~ max	6.70 – 32.00	7.40 – 32.20	7.80 – 32.10	6.70 – 32.20
Creatinine						
		n	393	393	393	1179
		mean ± Std	1.00 ± 0.18	1.00 ± 0.17	0.96 ± 0.18	0.99 ± 0.18
		Q1, Q3	0.85, 1.12	0.87, 1.11	0.82, 1.08	0.85, 1.11
		min ~ max	0.59 – 1.72	0.67 – 1.97	0.50 – 1.79	0.50 – 1.97
AST						
		n	393	393	393	1179
		mean ± Std	25.60 ± 7.18	24.41 ± 8.03	25.78 ± 11.99	25.26 ± 9.32
		Q1, Q3	21.00, 28.00	20.00, 27.00	21.00, 27.00	21.00, 27.00
		min ~ max	14.00 – 72.00	13.00 – 121.00	14.00 – 181.00	13.00 – 181.00
ALT						
		n	393	393	393	1179
		mean ± Std	24.48 ± 11.96	22.61 ± 10.27	24.08 ± 13.46	23.72 ± 11.98
		Q1, Q3	17.00, 28.00	16.00, 26.00	16.00, 27.00	16.00, 27.00
		min ~ max	6.00 – 118.00	8.00 – 74.00	10.00 – 158.00	6.00 – 158.00
Total_cholesterol						
		n	393	393	393	1179
		mean ± Std	200.81 ± 37.31	196.22 ± 33.81	193.37 ± 36.05	196.80 ± 35.85
		Q1, Q3	175.00, 226.00	173.00, 218.00	169.00, 215.00	172.00, 219.00
		min ~ max	116.00 – 330.00	109.00 – 313.00	95.00 – 341.00	95.00 – 341.00
HDL_cholesterol						
		n	393	393	393	1179
		mean ± Std	49.38 ± 12.86	47.58 ± 12.34	46.89 ± 12.53	47.95 ± 12.61
		Q1, Q3	40.00, 58.00	39.00, 55.00	38.00, 54.00	39.00, 55.00
		min ~ max	25.00 ~ 102.00	19.00 ~ 100.00	24.00 ~ 123.00	19.00 ~ 123.00

(Continued)

Supplementary Table 2. Continued

Variable	Category	Statistics	Phase 1 (N=393)	Phase 2 (N=393)	Phase 3 (N=393)	Total (N=1179)
Triglyceride						
		n	393	393	393	1179
		mean ± Std	136.87 ± 81.85	132.22 ± 81.01	130.86 ± 79.97	133.32 ± 80.92
		Q1, Q3	85.00, 162.00	84.00, 156.00	83.00, 151.00	84.00, 157.00
		min ~ max	34.00 – 878.00	37.00 – 901.00	35.00 – 714.00	34.00 – 901.00
CRP						
		n	393	393	393	1179
		mean ± Std	1.40 ± 4.46	1.30 ± 2.47	1.25 ± 2.52	1.32 ± 3.28
		Q1, Q3	0.39, 1.23	0.40, 1.12	0.37, 1.14	0.39, 1.15
		min ~ max	0.01 – 77.37	0.04 – 23.36	0.08 – 33.92	0.01 – 77.37
WBC						
		n	393	393	393	1179
		mean ± Std	5.15 ± 1.64	4.98 ± 1.43	5.30 ± 1.53	5.14 ± 1.54
		Q1, Q3	4.20, 5.80	4.00, 5.60	4.30, 5.90	4.20, 5.80
		min ~ max	2.10 – 22.80	2.30 – 12.50	2.00 – 17.90	2.00 – 22.80
RBC						
		n	393	393	393	1179
		mean ± Std	4.60 ± 0.44	4.60 ± 0.42	4.45 ± 0.40	4.55 ± 0.43
		Q1, Q3	4.29, 4.86	4.31, 4.91	4.17, 4.73	4.26, 4.85
		min ~ max	3.55 – 7.04	3.40 – 6.24	3.41 – 5.70	3.40 – 7.04
Hematocrit						
		n	393	393	393	1179
		mean ± Std	42.88 ± 4.25	43.67 ± 3.89	42.35 ± 3.86	42.97 ± 4.04
		Q1, Q3	40.10, 45.40	41.10, 46.40	39.50, 44.70	40.20, 45.60
		min ~ max	32.80 – 64.90	33.30 – 56.10	33.50 – 52.50	32.80 – 64.90
Platlet						
		n	393	393	393	1179
		mean ± Std	240.83 ± 55.77	237.89 ± 53.00	247.41 ± 57.25	242.04 ± 55.46
		Q1, Q3	201.00, 273.00	201.00, 273.00	212.00, 281.00	203.00, 275.00
		min ~ max	104.00 – 492.00	86.00 – 453.00	88.00 – 471.00	86.00 – 492.00

(Continued)

Supplementary Table 2. Continued

Variable	Category	Statistics	Phase 1 (N=393)	Phase 2 (N=393)	Phase 3 (N=393)	Total (N=1179)
Ins0						
		n	393	393	393	1179
		mean ± Std	8.33 ± 3.85	8.88 ± 4.05	9.14 ± 3.70	8.78 ± 3.88
		Q1, Q3	6.20, 9.30	6.40, 10.50	6.50, 11.20	6.35, 10.45
		min ~ max	1.60 – 33.00	1.60 – 29.10	2.10 – 25.90	1.60 – 33.00
Ins60						
		n	372	364	344	1080
		mean ± Std	53.37 ± 43.47	50.95 ± 37.41	34.65 ± 24.76	46.59 ± 37.19
		Q1, Q3	26.88, 63.90	26.18, 62.00	18.68, 42.05	24.00, 57.30
		min ~ max	2.40 – 345.90	2.50 – 248.40	2.10 – 185.40	2.10 – 345.90
Ins120						
		n	372	364	343	1079
		mean ± Std	50.94 ± 44.81	49.63 ± 42.07	41.41 ± 47.13	47.47 ± 44.82
		Q1, Q3	23.15, 61.10	19.70, 65.80	17.60, 49.75	20.20, 60.35
		min ~ max	1.60 – 343.00	2.40 – 227.00	1.00 – 670.00	1.00 – 670.00
BMI						
		n	393	393	393	1179
		mean ± Std	25.01 ± 2.93	24.87 ± 2.93	24.84 ± 3.01	24.90 ± 2.96
		Q1, Q3	23.25, 26.75	23.11, 26.72	23.03, 26.64	23.10, 26.72
		min ~ max	17.05 – 34.60	16.95 – 35.15	15.33 – 34.99	15.33 – 35.15

Descriptive statistics were presented for each KARE cohort phenotypes.

Supplementary Table 3. Associated SNPs used for Mendelian randomization analysis

RS number	CHR	Associated phenotype	Phenotype category	Beta	p-value
rs12531703	7	3-hydroxybutyrate	Metabolites	852.2124	9.19E-06
rs73452690	9	3-hydroxybutyrate	Metabolites	1278.7947	2.96E-07
rs2259835	12	3-hydroxybutyrate	Metabolites	1004.6325	1.31E-06
rs56934236	13	3-hydroxybutyrate	Metabolites	1814.7711	2.35E-06
rs4786179	16	3-hydroxybutyrate	Metabolites	1402.7483	2.32E-06
rs9939748	16	3-hydroxybutyrate	Metabolites	1755.9474	2.17E-07
rs201406727	17	3-hydroxybutyrate	Metabolites	1846.7954	2.64E-06
rs77883150	3	3-hydroxybutyrate	Metabolites	1820.5233	6.37E-07
rs12531703	7	acetoacetate	Metabolites	145.1785	1.44E-06
rs10234305	7	acetoacetate	Metabolites	206.6373	1.52E-06
rs11677140	2	acetoacetate	Metabolites	312.1457	5.50E-08
rs222001	4	acetoacetate	Metabolites	173.5119	3.75E-06
rs6829597	4	acetoacetate	Metabolites	145.7757	3.05E-06
rs954368	4	Relative proportion of *GU174097_g*	Metagenome	0.0017	3.92E-06
rs78545803	7	Relative proportion of *GU174097_g*	Metagenome	0.0026	4.83E-06
rs7042888	9	Relative proportion of *GU174097_g*	Metagenome	−0.0011	2.60E-06
rs12934989	16	Relative proportion of *GU174097_g*	Metagenome	0.0020	8.43E-07
rs12936791	17	Relative proportion of *GU174097_g*	Metagenome	0.0024	1.25E-07
rs2040202	18	Relative proportion of *GU174097_g*	Metagenome	0.0015	2.14E-06
rs118068889	3	Relative proportion of *GU174097_g*	Metagenome	0.0024	6.59E-06
rs1934826	13	Log of HbA1c	Clinical	−0.0139	1.88E-06
rs1190552	14	Log of HbA1c	Clinical	−0.0129	9.06E-06
rs113844473	18	Log of HbA1c	Clinical	0.0257	5.43E-06
rs17795663	5	Log of HbA1c	Clinical	0.0132	6.05E-06
rs6903175	6	Log of HbA1c	Clinical	−0.0142	1.00E-06

Supplementary Table 3. Continued

RS number	CHR	Associated phenotype	Phenotype category	Beta	p-value
rs1934826	13	Log of fasting glucose level	Clinical	−0.0196	2.10E-06
rs7574648	2	Log of fasting glucose level	Clinical	−0.0262	2.76E-06
rs10163368	16	Log of fasting glucose level	Clinical	0.0208	3.24E-06
rs12954376	18	Log of fasting glucose level	Clinical	0.0285	7.04E-06
rs4142050	20	Log of fasting glucose level	Clinical	0.0222	3.08E-07
rs11763596	7	Log of fasting glucose level	Clinical	0.0217	2.74E-06
rs12607963	18	Log of 60-min glucose level	Clinical	−0.0434	3.20E-06
rs7820960	8	Log of 60-min glucose level	Clinical	−0.0411	8.95E-06
rs57643685	11	Log of 120-min glucose level	Clinical	0.0436	3.97E-06
rs80071180	17	Log of 120-min glucose level	Clinical	−0.0694	2.57E-06
rs4941100	18	Log of 120-min glucose level	Clinical	−0.0396	1.33E-06
rs6739228	2	Log of 120-min glucose level	Clinical	−0.0513	9.08E-07
rs201894669	7	Log of 120-min glucose level	Clinical	0.0355	5.18E-06
rs12922338	16	Log of fasting insulin level	Clinical	0.0777	5.29E-06
rs17604444	17	Log of fasting insulin level	Clinical	−0.0436	1.31E-06
rs61777670	1	Log of fasting insulin level	Clinical	0.0816	3.28E-06

Supplementary Table 3. Continued

RS number	CHR	Associated phenotype	Phenotype category	Beta	p-value
rs17783505	12	Log of 60-min insulin level	Clinical	−0.0946	7.77E-06
rs2472660	7	Log of 60-min insulin level	Clinical	0.0721	4.50E-06
rs35244292	15	Log of 120-min insulin level	Clinical	0.1201	1.49E-06
rs1971134	2	Log of 120-min insulin level	Clinical	−0.0789	8.42E-06
rs77231286	3	Log of 120-min insulin level	Clinical	0.1882	3.50E-06
rs4258093	9	Log of 120-min insulin level	Clinical	−0.0811	7.77E-06
rs6604867	1	Log of BMI	Clinical	−0.0219	1.20E-06
rs7481015	11	Log of Waist Hip Ratio	Clinical	0.0063	8.16E-06
rs1343820	12	Log of Waist Hip Ratio	Clinical	−0.0083	2.47E-06
rs80073529	2	Log of Waist Hip Ratio	Clinical	0.0065	6.86E-06

Supplementary Table 4. Association analysis of type 2 diabetes (T2D) with functional profiles predicted using Tax4Fun

Phenotype	KEGG Pathway	Estimate	StdErr	DF	*p*-value	FDR
T2D-at-risk/T2D	*Cationic antimicrobial peptide (CAMP) resistance*	−105.77	28.99	920	0.00028	0.03735
Binary_T2D	*Bacterial secretion system*	−104.43	34.02	920	0.00221	0.09027
Binary_T2D	*Base excision repair*	−236.06	79.69	920	0.00313	0.09027
Binary_T2D	*Taurine and hypotaurine metabolism*	378.99	129.15	920	0.00342	0.09027
Binary_T2D	*Glycerophospholipid metabolism*	−192.29	66.15	920	0.00374	0.09027
Binary_T2D	*Pantothenate and CoA biosynthesis*	−114.77	39.82	920	0.00404	0.09027
Binary_T2D	*Fatty acid biosynthesis*	75.86	27.60	920	0.00611	0.09528
Binary_T2D	*beta-Lactam resistance*	−65.95	24.46	920	0.00715	0.09528
Binary_T2D	*Oxidative phosphorylation*	−91.71	34.08	920	0.00726	0.09528
Binary_T2D	*Biosynthesis of secondary metabolites*	−22.08	8.32	920	0.00806	0.09528

Associations of functional profiles with *T2D-at-ristk/T2D* and *Binary_T2D* were tested and significant associations at FDR-adjusted 0.1 significance level were summarized.

Supplementary Table 5. Association analysis of genera with diabetes risk indicators

Phenotype	Genus	Estimate	p-value	FDR
Ins120	*Diaphorobacter*	8.7235	4.32E-06	0.000302
Hba1c	*Fusobacterium*	−0.6144	5.30E-06	0.000371
Hba1c	*Gardnerella*	−0.2588	3.31E-05	0.000772
Hba1c	*Hafnia*	0.1691	2.59E-05	0.000772
Hba1c	*Akkermansia*	−0.08445	4.69E-05	0.000822
Ins0	*Bacteroides*	−0.563	1.89E-05	0.001325
Glu60	*Faecalibacterium*	1.7566	2.37E-05	0.001351
Ins120	*Mucispirillum*	4.6197	4.77E-05	0.001668
Glu0	*AB185816_g*	1.9868	3.25E-05	0.002274
Ins60	*Paracoccus*	−3.1448	0.000117	0.008211
Hba1c	*AB185816_g*	1.184	0.000591	0.008271
Ins60	*Akkermansia*	0.8599	0.000644	0.014056
Ins60	*Bacillus*	−10.4746	0.000525	0.014056
Ins60	*Hafnia*	−2.1636	0.000803	0.014056
Ins60	*Eubacterium_g23*	3.3619	0.001052	0.014733
Glu0	*Gardnerella*	−0.2234	0.000428	0.014985
Hba1c	*KE159538_g*	−0.8552	0.001459	0.017027
Ins120	*Hafnia*	−2.996	0.000824	0.019217
Ins60	*Bifidobacterium*	−2.2198	0.001773	0.020682
Ins60	*AF349416_g*	11.2469	0.003463	0.030304
Ins60	*Clostridium*	2.7748	0.003281	0.030304
Ins60	*EU622770_g*	6.3967	0.005	0.03889
Ins60	*Subdoligranulum*	−4.5438	0.005617	0.039319
Glu0	*Akkermansia*	−0.08067	0.001739	0.040583
Ins60	*AB185816_g*	−13.129	0.008131	0.047429
Ins60	*Parabacteroides*	4.5801	0.008032	0.047429
Hba1c	*Collinsella*	0.182	0.005517	0.048343
Hba1c	*Pseudoflavonifractor*	−0.7954	0.005525	0.048343
Ins0	*Acinetobacter*	0.532	0.001467	0.050619
Ins0	*Fusobacterium*	2.5293	0.002169	0.050619
Hba1c	*Prevotella*	0.3263	0.008377	0.065156
Hba1c	*Agathobacter*	0.1971	0.014171	0.082664
Hba1c	*Bifidobacterium*	0.1111	0.012478	0.082664
Hba1c	*Faecalibacterium*	0.2225	0.013039	0.082664
Ins120	*Oscillibacter*	7.0022	0.004943	0.086495
Ins60	*Methanobrevibacter*	−3.4821	0.017655	0.095066

Associations of genera with diabetes-risk indicators were tested and significant associations at FDR-adjusted 0.1 significance level were summarized.

Supplementary Table 6. One-sample Mendelian randomization causal effect

Exposure	Outcome	Weak Instrument *p*-value	DWH *p*-value	Estimate (95% CI)	*p*-value
GU174097_g	3-hydroxybutyrate	1.8E-05	0.334	-3.6E-07 (-9.2E-07, 1.9E-07)	0.196
GU174097_g	Acetoacetate	8.8E-04	0.264	-2.4E-06 (-6.0E-06, 1.2E-06)	0.193
3-hydroxybutyrate	*GU174097_g*	1.1E-07	0.495	-1.4E+05 (-4.1E+05, 1.3E+05)	0.297
Acetoacetate	*GU174097_g*	1.1E-07	0.488	-1.9E+04 (-5.9E+04, 2.1E+04)	0.353

Two stage least squares is used for one-sample mendelian randomization. Weak Instrument *p*-value checks weak instrument bias and DWH *p*-value checks the endogeneity.

Supplementary Table 7. Statistical analysis to check the assumption required for two-sample Mendelian randomization

Outcome	*GU174097_g*					**3-hydroxybutyrate**					**Acetoacetate**				
	***N*=7 *F*=10.45**					***N*=8 *F*=16.23**					***N*=5 *F*=17.77**				
	I^2	***Q***	***Q′***	***RSS***	**Suggested MR method**	I^2	***Q***	***Q′***	***RSS***	**Suggested MR method**	I^2	***Q***	***Q′***	***RSS***	**Suggested MR method**
HbA1c	0*	0.533	0.644	0.651	IVW	0*	0.703	0.694	0.693	IVW	0*	0.424	0.014*	0.012*	MR-Egger (SIMEX)
Glu0	0*	0.372	0.181	0.183	IVW	0*	0.765	0.851	0.852	IVW	0*	0.819	0.157	0.121	IVW
Glu60	0*	0.660	0.686	0.694	IVW	0*	0.536	0.643	0.644	IVW	0*	0.222	0.320	0.347	IVW
Glu120	0*	0.355	0.457	0.473	IVW	0*	0.461	0.435	0.443	IVW	0*	0.105	0.104	0.119	IVW
Ins0	0*	0.542	0.668	0.666	IVW	0*	0.694	0.794	0.787	IVW	0*	0.913	0.691	0.645	IVW
Ins60	0*	0.117	0.174	0.184	IVW	0*	0.213	0.050	0.048*	MR-Egger (SIMEX)	0*	0.627	0.762	0.781	IVW
Ins120	0*	0.463	0.257	0.271	IVW	0*	0.924	0.902	0.897	IVW	0*	0.688	0.707	0.717	IVW
BMI	0*	0.758	0.840	0.846	IVW	0*	0.165	0.241	0.257	IVW	0*	0.301	0.198	0.226	IVW

Q, heterogeneity test from IVW; *Q′*, *P*-value for Cochran's Q test; *RSS*, *P* for MR-PRESSO global test, *F*, mean F statistic; I^2, I^2 value from MR-Egger. *F*-test checks weak instrument bias, I^2 checks the NOME assumption and *Q* and *Q′*, and *RSS* checks the heterogeneity assumption.

Supplementary Table 8. Detection of SNPs that potentially cause pleiotropy.

The result of outlier tests of MR-PRESSO are presented for the associated SNPs where heterogeneity was detected. SNPs with significant *p*-value indicates that they can produce horizontal pleiotropy.

RS number	CHR	Associated exposure	Response	RSSobs	p-value
rs12531703	7	acetoacetate	Log of HbA1c	2.46E-06	1
rs10234305	7	acetoacetate	Log of HbA1c	1.49E-05	1
rs11677140	2	acetoacetate	Log of HbA1c	3.40E-04	0.0728
rs222001	4	acetoacetate	Log of HbA1c	3.70E-05	1
rs6829597	4	acetoacetate	Log of HbA1c	1.18E-04	0.0566
rs12531703	7	3-hydroxybutyrate	Log of Ins60	5.59E-06	1
rs73452690	9	3-hydroxybutyrate	Log of Ins60	2.10E-05	1
rs2259835	12	3-hydroxybutyrate	Log of Ins60	3.82E-03	0.0122
rs56934236	13	3-hydroxybutyrate	Log of Ins60	6.15E-04	1
rs4786179	16	3-hydroxybutyrate	Log of Ins60	5.05E-05	1
rs9939748	16	3-hydroxybutyrate	Log of Ins60	2.43E-03	0.789
rs201406727	17	3-hydroxybutyrate	Log of Ins60	2.87E-03	0.7166
rs77883150	3	3-hydroxybutyrate	Log of Ins60	3.84E-04	1

Supplementary Table 9. Two-sample Mendelian randomization causal effect

Outcome	MR methods	*GU174097_g*		3-hydroxybutyrate		Acetoacetate	
		Estimate (95% CI)	FDR	Estimate (95% CI)	FDR	Estimate (95% CI)	FDR
HbA1c	Weighted Median	0.632 (−1.7011, 2.965)	0.595	9.5e−07 (−2.1e−06, 4e−06)	0.595	3e−05 (3.3e−07, 5.9e−05)	0.143
	MR-Egger	−0.997 (−7.212, 5.2179)	0.753	−3.3e−06 (−1.2e−05, 5.3e−06)	0.677	0.00013 (6.2e−05, 0.0002)	0.001
	MR-Egger (SIMEX)	0.7775 (−1.0014, 2.5564)	0.431	−4.6e−06 (−1.4e−05, 4.4e−06)	0.431	**0.00016 (9.8e−05, 0.00022)**	**0.046***
	IVW	**0.0695 (−1.7213, 1.8604)**	**0.939**	**7.4e−07 (−1.6e−06, 3.1e−06)**	**0.810**	2.5e−05 (−1.1e−05, 6e−05)	0.533
	MR-PRESSO	0.0695 (−1.436, 1.5751)	0.931	7.4e−07 (−1.2e−06, 2.7e−06)	0.720	2.5e−05 (−1.1e−05, 6e−05)	0.720
Glu0	Weighted Median	−0.0985 (−3.5584, 3.3613)	0.955	2.1e−06 (−2.1e−06, 6.3e−06)	0.496	2.9e−05 (−1e−05, 6.8e−05)	0.438
	MR−Egger	−7.8089 (−16.9558, 1.338)	0.141	1.6e−06 (−1.1e−05, 1.4e−05)	0.794	0.00015 (4.8e−05, 0.00025)	0.011
	MR−Egger (SIMEX)	2.4721 (−0.1467, 5.0909)	0.185	2.3e−06 (−9.7e−06, 1.4e−05)	0.717	0.00018 (0.00016, 0.0002)	0.001
	IVW	**0.2604 (−2.8322, 3.353)**	**0.869**	**1.3e−06 (−2.1e−06, 4.6e−06)**	**0.679**	**3.1e−05 (−5.8e−06, 6.8e−05)**	**0.295**
	MR−PRESSO	0.2604 (−2.8322, 3.353)	0.874	1.3e−06 (−1e−06, 3.6e−06)	0.470	3.1e−05 (−5.8e−06, 6.8e−05)	0.470
Glu60	Weighted Median	−2.7662 (−8.5932, 3.0608)	0.919	−1.1e−06 (−8.6e−06, 6.5e−06)	0.919	−3.4e−06 (−6.9e−05, 6.3e−05)	0.919
	MR-Egger	−8.4801 (−24.0515, 7.0914)	0.658	−5.7e−06 (−2.7e−05, 1.6e−05)	0.658	4.9e−05 (−0.00017, 0.00027)	0.658
	MR-Egger (SIMEX)	−1.5038 (−6.3334, 3.3259)	0.708	−7.8e−06 (−3.4e−05, 1.9e−05)	0.708	5.7e−05 (−0.00022, 0.00033)	0.708
	IVW	**−2.2526 (−6.749, 2.2438)**	**0.530**	**−2.8e−06 (−8.7e−06, 3.1e−06)**	**0.530**	**5.9e−07 (−5.5e−05, 5.6e−05)**	**0.983**
	MR−PRESSO	−2.2526 (−5.8922, 1.387)	0.471	−2.8e−06 (−7.8e−06, 2.3e−06)	0.471	5.9e−07 (−5.5e−05, 5.6e−05)	0.984
Glu120	Weighted Median	−3.1981 (−9.4609, 3.0647)	0.490	−2.9e−06 (−1.1e−05, 5.3e−06)	0.490	−2.5e−05 (−9.6e−05, 4.6e−05)	0.490
	MR-Egger	−7.5848 (−24.8455, 9.6759)	0.455	−1.5e−05 (−3.8e−05, 7.7e−06)	0.455	0.0001 (−0.00017, 0.00038)	0.455
	MR-Egger (SIMEX)	−3.7682 (−10.031, 2.4945)	0.437	−2.1e−05 (−5e−05, 7.3e−06)	0.437	0.00012 (−0.00022, 0.00047)	0.530
	IVW	**−4.2316 (−8.9665, 0.5034)**	**0.240**	**−2.5e−06 (−8.7e−06, 3.7e−06)**	**0.653**	**−1.2e−05 (−8.6e−05, 6.3e−05)**	**0.761**
	MR−PRESSO	−4.2316 (−8.8492, 0.3861)	0.368	−2.5e−06 (−8.7e−06, 3.7e−06)	0.689	−1.2e−05 (−8.6e−05, 6.3e−05)	0.777

The recommended MR method is highlighted in bold letters.

Supplementary Table 9. Continued

Outcome	MR methods	*GU174097_g*		3-hydroxybutyrate		Acetoacetate	
		Estimate (95% CI)	FDR	Estimate (95% CI)	FDR	Estimate (95% CI)	FDR
Ins0	Weighted Median	0.0809 (−6.646, 6.8077)	0.981	−1e−07 (−8.7e−06, 8.5e−06)	0.981	1.7e−05 (−5.3e−05, 8.8e−05)	0.981
	MR-Egger	0.1588 (−17.656, 17.9735)	0.986	−1e−06 (−2.6e−05, 2.4e−05)	0.986	0.00014 (−6.1e−05, 0.00034)	0.517
	MR-Egger (SIMEX)	3.0289 (−2.0456, 8.1034)	0.442	−1.3e−06 (−2.8e−05, 2.5e−05)	0.928	0.00017 (8.4e−05, 0.00025)	0.091
	IVW	**1.1125 (−4.0195, 6.2444)**	**0.829**	**−7.4e−07 (−7.4e−06, 6e−06)**	**0.829**	**1.1e−05 (−4.6e−05, 6.9e−05)**	**0.829**
	MR−PRESSO	1.1125 (−3.1119, 5.3368)	0.780	−7.4e−07 (−5.7e−06, 4.2e−06)	0.780	1.1e−05 (−3.2e−05, 5.4e−05)	0.780
Ins60	Weighted Median	7.518 (−6.0625, 21.0984)	0.468	9e−06 (−8.4e−06, 2.6e−05)	0.468	−2e−05 (−0.00015, 0.00011)	0.763
	MR-Egger	10.0106 (−33.5852, 53.6064)	0.787	6e−05 (6.1e−06, 0.00011)	0.087	−5.3e−05 (−0.00043, 0.00033)	0.787
	MR-Egger (SIMEX)	8.2196 (−5.8634, 22.3025)	0.457	**8.4e−05 (1.8e−05, 0.00015)**	**0.143**	−6.5e−05 (−0.00042, 0.00029)	0.741
	IVW	**3.2629 (−8.3365, 14.8623)**	**0.859**	6.6e−06 (−1.1e−05, 2.4e−05)	0.859	**9.7e−06 (−9.8e−05, 0.00012)**	**0.859**
	MR−PRESSO	3.2629 (−8.3365, 14.8623)	0.807	1.4e−05 (3.5e−06, 2.5e−05)	0.121	9.7e−06 (−6.3e−05, 8.3e−05)	0.807
Ins120	Weighted Median	−11.4001 (−26.8887, 4.0885)	0.447	−3e−07 (−1.8e−05, 1.8e−05)	0.988	−1.2e−06 (−0.00015, 0.00015)	0.988
	MR-Egger	24.9815 (−13.0067, 62.9696)	0.399	2.9e−05 (−2.4e−05, 8.1e−05)	0.399	2e−04 (−3e−04, 6e−04)	0.399
	MR-Egger (SIMEX)	−11.541 (−27.7749, 4.6929)	0.324	4e−05 (1.1e−06, 7.9e−05)	0.272	2e−04 (−2e−04, 6e−04)	0.324
	IVW	**−7.7912 (−20.2658, 4.6835)**	**0.663**	**4.5e−06 (−9.9e−06, 1.9e−05)**	**0.812**	**1.1e−05 (−0.00011, 0.00014)**	**0.860**
	MR−PRESSO	−7.7912 (−20.2658, 4.6835)	0.551	4.5e−06 (−4.6e−06, 1.4e−05)	0.551	1.1e−05 (−8e−05, 0.0001)	0.822
BMI	Weighted Median	0.5582 (−1.772, 2.8884)	0.639	1.3e−06 (−1.9e−06, 4.4e−06)	0.639	2.2e−05 (−5.4e−06, 4.9e−05)	0.349
	MR-Egger	−1.1856 (−7.5103, 5.139)	0.890	−7.6e−07 (−1.2e−05, 1e−05)	0.890	6.5e−05 (−1.4e−05, 0.00015)	0.324
	MR-Egger (SIMEX)	0.4651 (−0.9768, 1.907)	0.833	−1.1e−06 (−1.5e−05, 1.3e−05)	0.882	7.7e−05 (−1.5e−05, 0.00017)	0.603
	IVW	**−0.1097 (−1.9344, 1.715)**	**0.906**	**−9.1e−07 (−3.6e−06, 1.8e−06)**	**0.773**	**1.1e−05 (−1.4e−05, 3.6e−05)**	**0.773**
	MR−PRESSO	−0.1097 (−1.3438, 1.1245)	0.867	−9.1e−07 (−3.6e−06, 1.8e−06)	0.805	1.1e−05 (−1.4e−05, 3.6e−05)	0.805

The recommended MR method is highlighted in bold letters.

Supplementary Fig. 1. Description of the genome-wide association studies (GWASs)

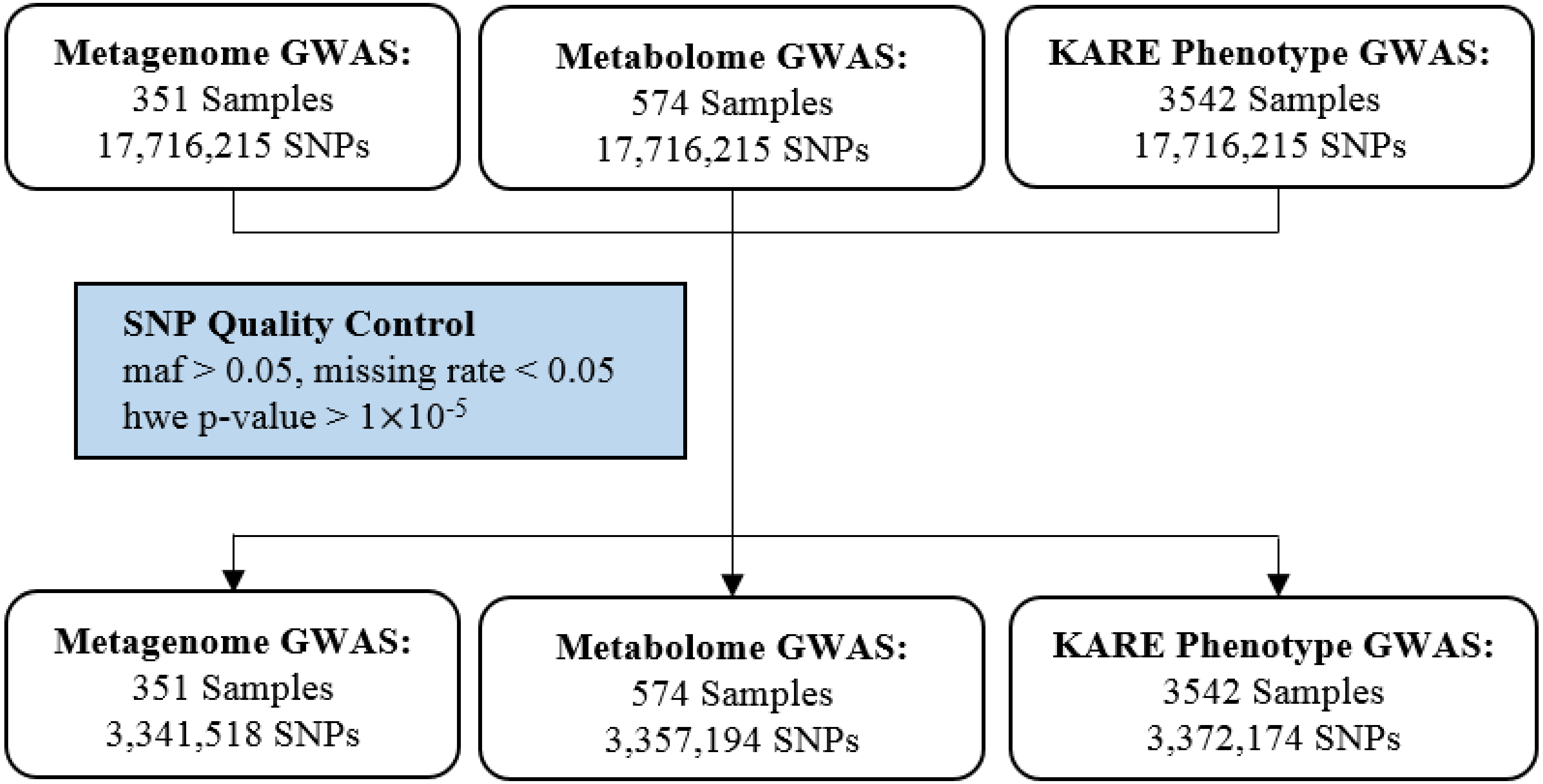

After individual quality control for KARE genomic data, 8,216 subjects with 17,716,215 SNP remained, and subjects for each GWAS were selected from these.

Supplementary Fig. 2. Box plots of alpha diversity indices for phases 1, 2, and 3.

The operational taxonomic unit (OTU) count table is rarefied and used for the calculation of Ace (**a**), Chao 1 (**b**), Shannon (**c**), and Simpson (d) indices.

a. ACE index

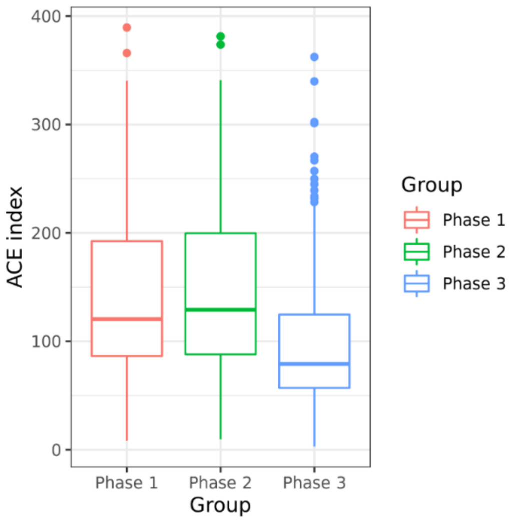

b. Chao1 index

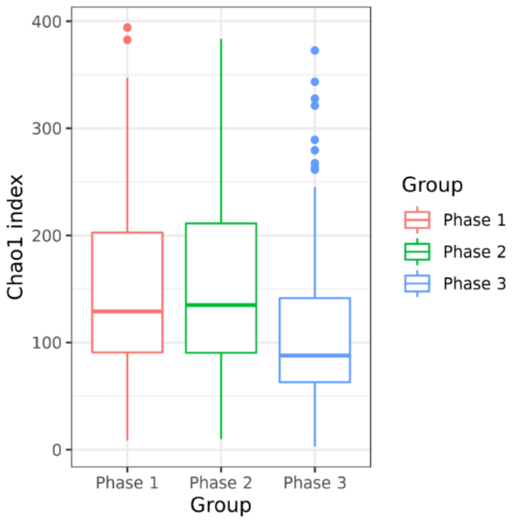

c. Shannon index

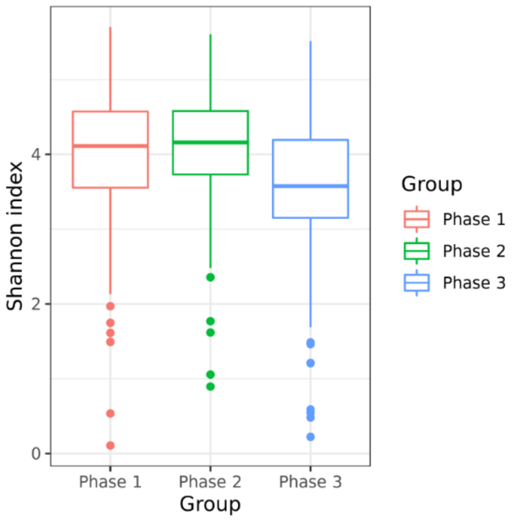

d. Simpson index

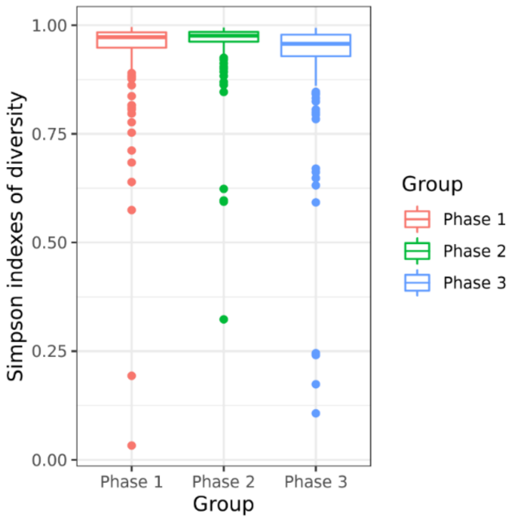

Supplementary Fig. 3. NMDS plot for phases 1, 2, and 3.

Bray–Curtis beta diversities based on urine samples for phase 1, 2, and 3.

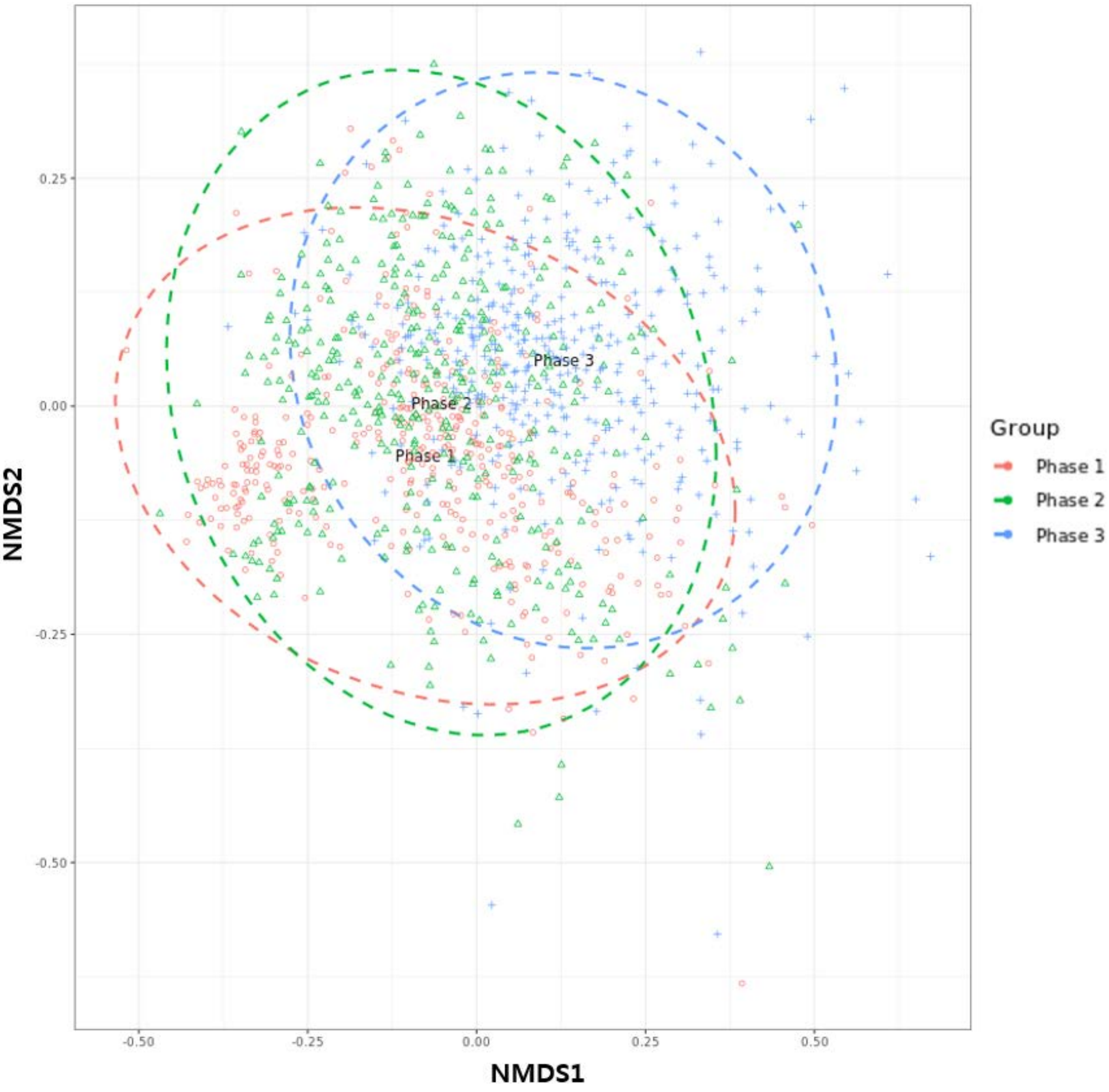

Supplementary Fig. 4. Taxonomic composition at the genus level for phases 1, 2, and 3.

Profile plots for phases 1, 2, and 3 showing the bacterial genera with the top 12 mean relative abundances.

a. Phase 1

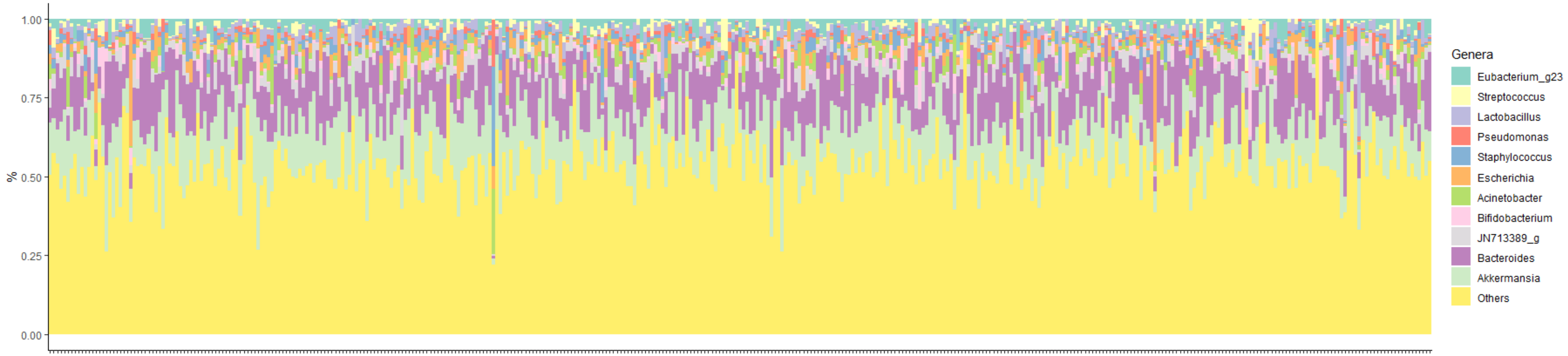

b. Phase 2

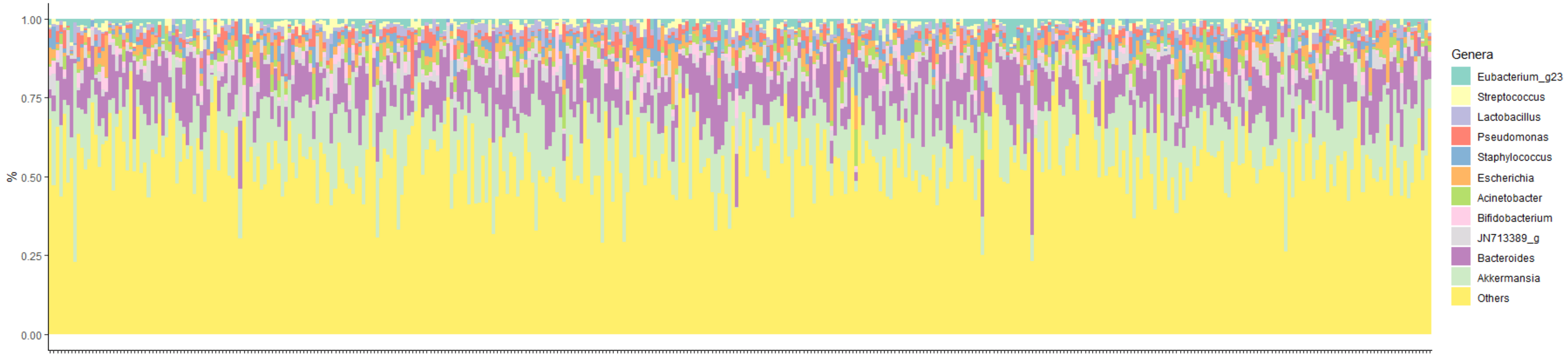

c. Phase 3

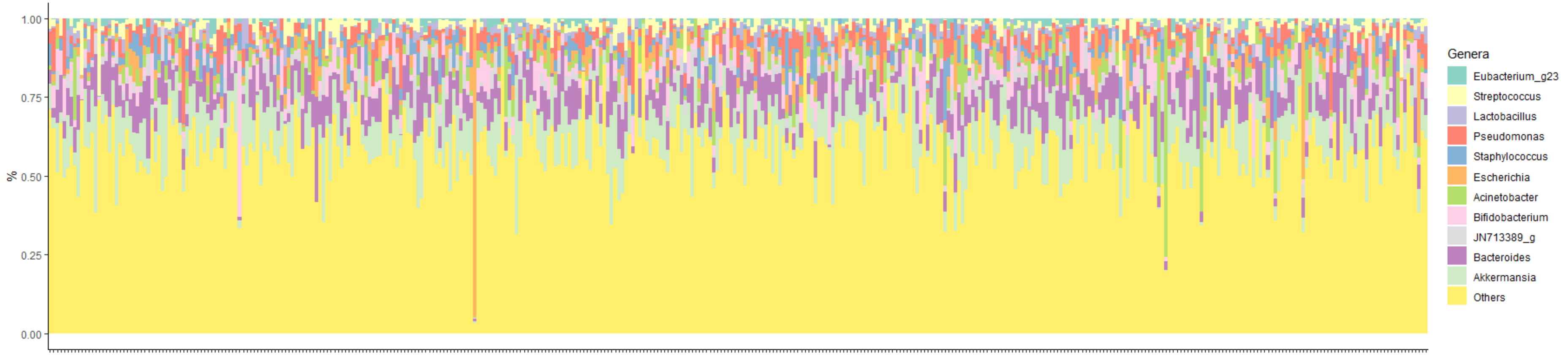
1.00
0.75
0.50
0.25
0.00
%
Genera
Eubacterium_g23
Streptococcus
Lactobacillus
Pseudomonas
Staphylococcus
Escherichia
Acinetobacter
Bifidobacterium
JN713389_g
Bacteroides
Akkermansia
Others

Supplementary Fig. 5. Relative importance of variables.

The relative proportions of variance attributable to each variable were calculated with PERMANOVA using pldist based on Bray–Curtis beta-diversity. Every trait was categorized into four groups, including pre-defined diabetes-risk indicators, general information (age and sex), T2D outcomes (*Binary_T2D and T2D-at-risk/T2D*), and others.

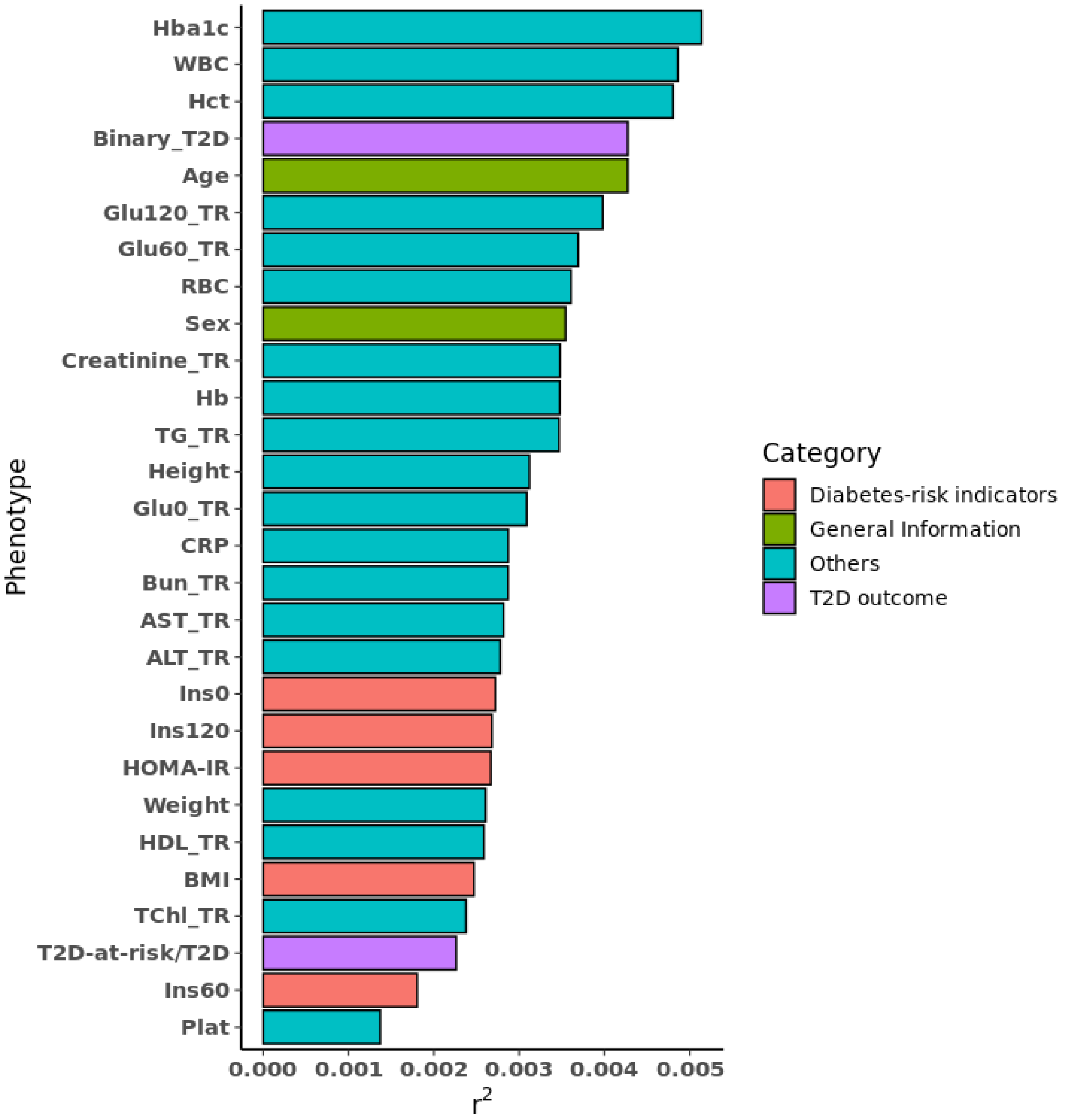

Supplementary Fig. 6. Changes in bacterial composition of each individual in type 2 diabetes (T2D) groups.

Changes of bacterial composition were described for all the individuals in the *T2D on Phase 1-3*, *Healthy on Phase 1-3*, and *T2D-at-risk/T2D to Healthy* groups. For the *Healthy on Phase 1-3* group, 20 subjects were randomly subsampled for readability of the plot.

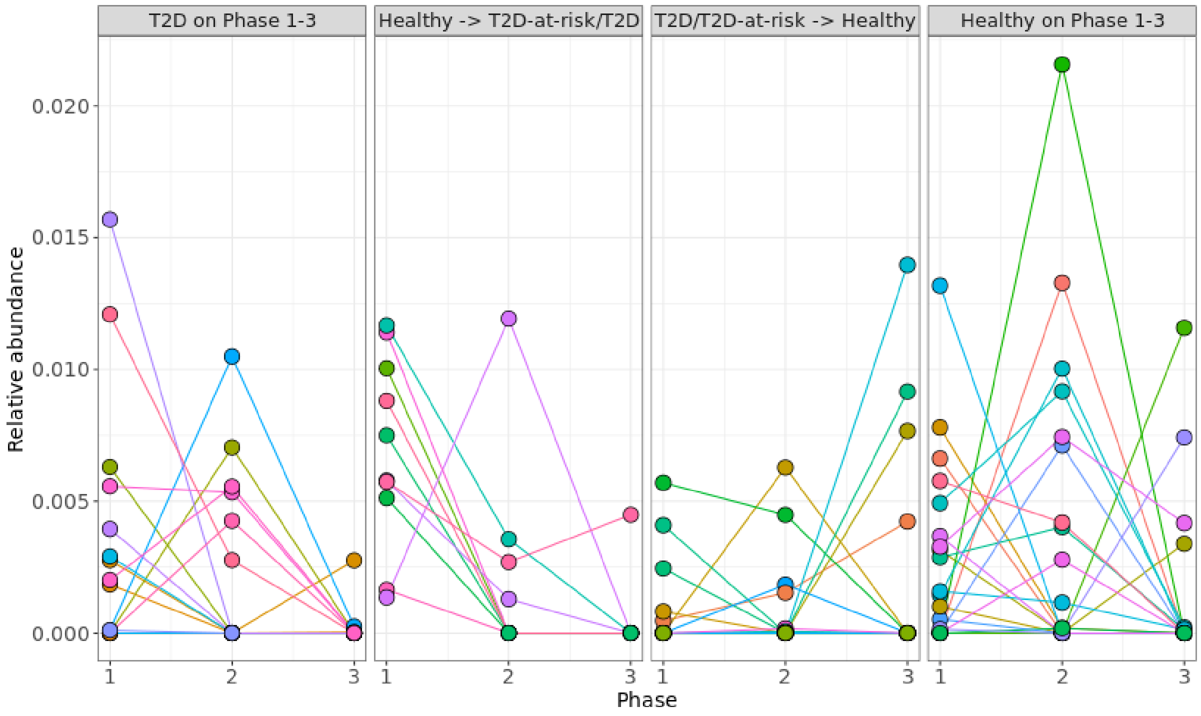

Supplementary Fig. 7. Correlation between *GU174097_g* and each ketone body type.

Serial correlations were removed with using residuals of *GU174097*_g, acetoacetate, and 3-hydroxybutyrate based on the linear mixed model, with age and sex used as covariates.

a. Acetoacetate

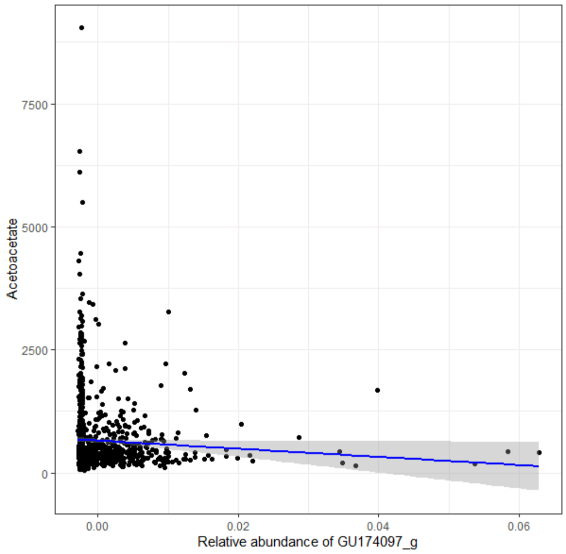

b. 3-hydroxybutyrate

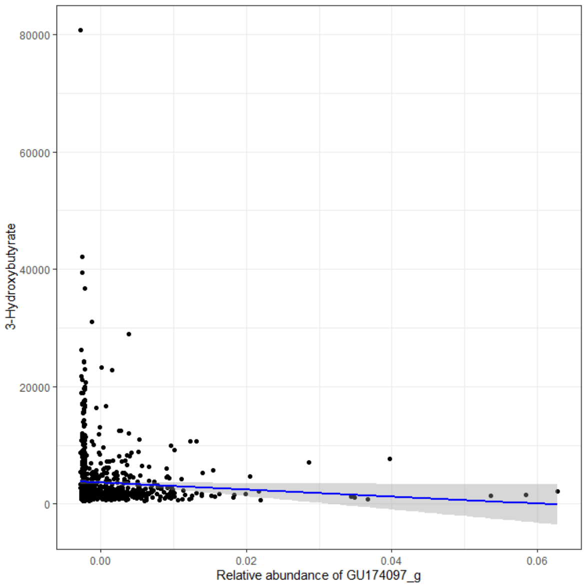

Supplementary Fig. 8 Network of *GU174097_g* and KARE phenotypes

At FDR < 0.05, edge width is in bold; otherwise, edge width is not included. When rho is positive, edges are colored red; otherwise, they are colored blue. Blue, green, and red nodes represent KARE phenotypes, *GU174097_g*, and Homeostatic Model Assessment for Insulin Resistance (HOMA-IR), respectively.

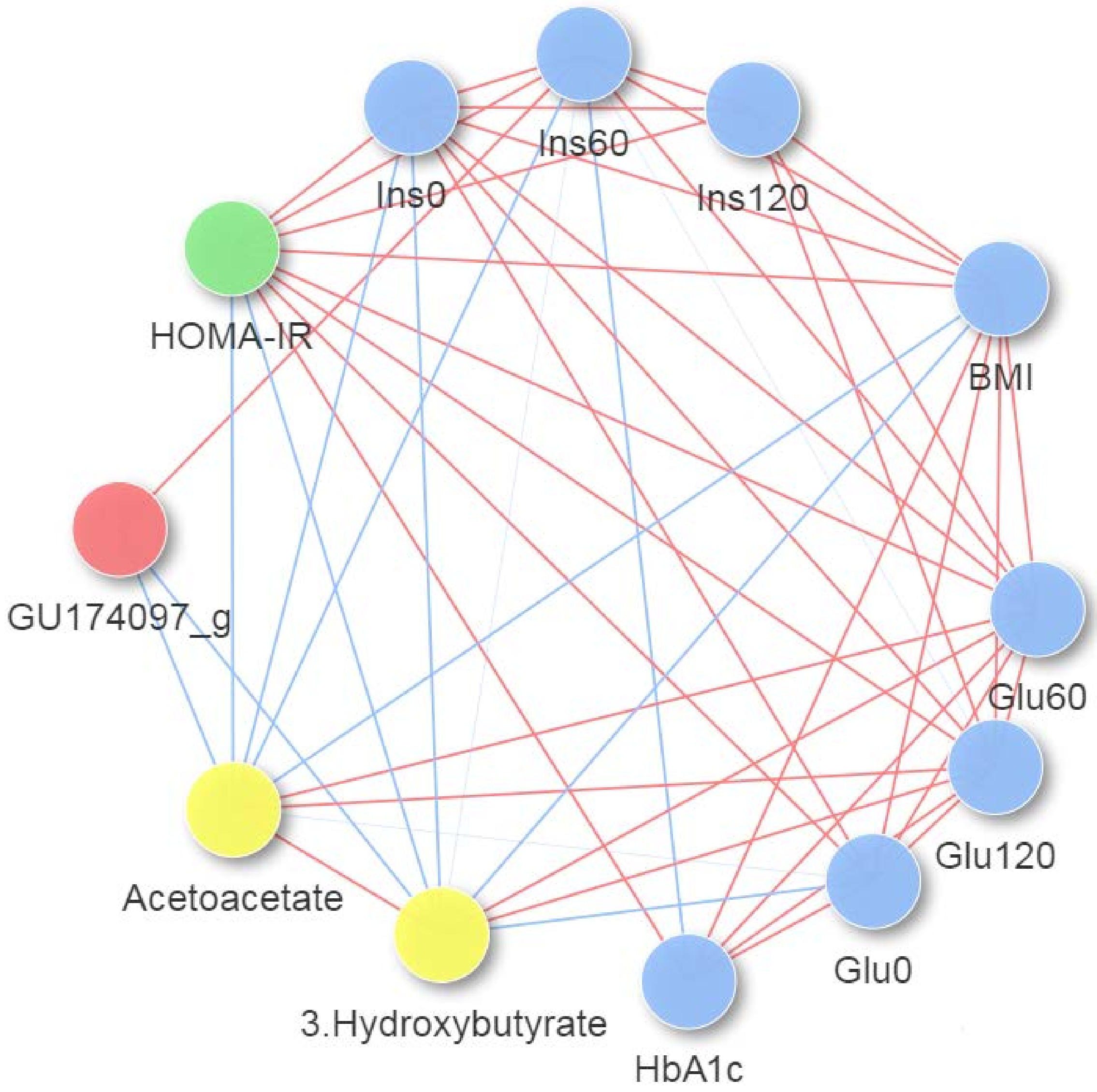

Supplement: Supplementary file 1 — Supplementary information [file 12276_2022_816_MOESM1_ESM.pdf]
